# A combined NMR and deep neural network approach for enhancing the spectral resolution of aromatic side chains in proteins

**DOI:** 10.1126/sciadv.adr2155

**Published:** 2024-12-20

**Authors:** Vaibhav Kumar Shukla, Gogulan Karunanithy, Pramodh Vallurupalli, D. Flemming Hansen

**Affiliations:** ^1^ Department of Structural and Molecular Biology, Division of Biosciences, University College London, London WC1E 6BT, UK.; ^2^ Tata Institute of Fundamental Research Hyderabad, 36/P, Gopanpally Village, Serilingampally Mandal, Ranga Reddy District, Hyderabad 500046, India.; ^3^ The Francis Crick Institute, London NW1 1AT, UK.

## Abstract

Nuclear magnetic resonance (NMR) spectroscopy is an important technique for deriving the dynamics and interactions of macromolecules; however, characterizations of aromatic residues in proteins still pose a challenge. Here, we present a deep neural network (DNN), which transforms NMR spectra recorded on simple uniformly ^13^C-labeled samples to yield high-quality ^1^H-^13^C correlation maps of aromatic side chains. Key to the success of the DNN is the design of NMR experiments that produce data with unique features to aid the DNN produce high-resolution spectra. The methodology was validated experimentally on protein samples ranging from 7 to 40 kDa in size, where it accurately reconstructed multidimensional aromatic ^1^H-^13^C correlation maps, to facilitate ^1^H-^13^C chemical shift assignments and to quantify kinetics. More generally, we believe that the strategy of designing new NMR experiments in combination with customized DNNs represents a substantial advance that will have a major impact on the study of molecules using NMR in the years to come.

## Teaser

Combining developments in deep learning and NMR opens up opportunities like high-resolution aromatic spectra from protein samples.

## INTRODUCTION

Nuclear magnetic resonance (NMR) spectroscopy is a ubiquitous technique in material science, chemistry, structural biology, and clinical diagnosis. In bioscience, NMR provides unprecedented insight into functional motions (*1*–*7*) and noncovalent interactions (*8*–*10*) with atomic resolution. The technique therefore excellently complements artificial intelligence (AI)–generated protein structures, e.g., from AlphaFold2, as well as structures obtained by cryo–electron microscopy (*11*–*13*).

Over many decades, a series of developments that include advances in hardware, sample preparation, and NMR pulse sequences have steadily raised the “size limits” of proteins that can be studied using solution-state NMR. Specific advances include the introduction of per-deuteration (*14*), ^15^N-^1^H TROSY (*15*), and methyl-TROSY methods (*16*). Using these techniques, it is now possible to record amide ^15^N-^1^H and methyl ^13^C-^1^H correlation maps in megadalton-sized proteins. However, studying functional side chains, such as charged or aromatic side chains, which are often present in enzymatic active sites and within interaction hotspots, are much more challenging.

We showed recently that using ^13^C-detection allows for a characterization of charged side chains, such as arginine and lysine, in proteins up to ~40 kDa (*17*, *18*). For small proteins, ^1^H-detected NMR methods are available to probe lysine and negatively charged side chains, which have provided insight into molecular recognition, salt bridge, and hydrogen-bond formations (*19*, *20*). These experiments are often performed on uniformly ^13^C-labeled proteins samples using constant-time (CT) experiments that eliminate the peak splitting arising due to homonuclear ^1^*J*_CC_ couplings in the indirect ^13^C dimension (*21*, *22*) to record high resolution [^13^C-^1^H] correlation maps at different backbone and side-chain sites.

Characterization of aromatic side chains, on the other hand, has generally required specific labeling (*23*–*26*) because of nonuniform ^1^*J*_CC_ couplings and attenuation of signal due to substantial transverse relaxation during the CT period. There is therefore a clear need for improved methods to facilitate more detailed analysis of aromatic residues and their dynamics within proteins over a range of sizes of proteins to promote a greater understanding of how proteins function and interact.

Deep learning methods have had a substantial impact on all areas of science in recent years (*27*), solving key problems in biophysics and computational biology (*13*, *28*). Previous work from us and others have demonstrated applications of deep neural networks (DNNs) for transforming and analyzing magnetic resonance data including analyzing EPR DEER data (*29*), reconstructing nonuniformly sampled spectra, peak picking, and virtual homonuclear decoupling (*30*–*34*). Key to the success of these networks has been the ability to simulate an arbitrary amount of realistic training data (*29*, *34*), overcoming problems of overfitting and data bottlenecks that often beset these models. A shortcoming that exists in many existing DNNs in the field, however, is their inability to report reliable and quantitative uncertainties associated with the transformations.

In this work, we present a DNN architecture, FID-Net-2, which uses data from a specially designed set of NMR experiments to not only reconstruct high-resolution ^1^H-^13^C correlation maps of the aromatic side chains in proteins but also provide the uncertainty associated with the resulting spectra. The correlation maps generated by the DNN are free of the multiplet splittings and line broadenings that traditionally have degraded the quality of such spectra. We have validated this DNN-based methodology experimentally by accurately reconstructing high-resolution aromatic ^1^H-^13^C correlation spectra of the ~20-kDa L99A mutant of T4 lysozyme (L99A-T4L) and the 40-kDa maltose binding protein (MBP). Further, the utility of this methodology is demonstrated by (i) reconstructing high-resolution three-dimensional (3D) aromatic-methyl NOESY spectra to obtain aromatic ^1^H-^13^C assignments and (ii) quantitating the peak intensities in the reconstructed high-resolution aromatic ^1^H-^13^C correlation maps recorded with varying exchange times to obtain the forward and reverse rate constants for the folding of the A39G mutants of the FF domain (A39G-FF) from human HYPA/FBP11.

## RESULTS

Because of variable ^1^*J*_CC_ couplings (~55 to ~72 Hz) and fast ^13^C transverse relaxation, CT experiments are not routinely used to record high resolution ^13^C-^1^H correlation maps at various aromatic sites in proteins. Hence, we decided to develop a DNN to transform regular HSQC-like spectra, which contain multiplet splittings in the indirect (^13^C) dimension, into a high-resolution ^13^C-^1^H correlation map with sharp singlet peaks in the ^13^C dimension.

### Designing a pulse sequence to aid recognition of the aromatic multiplet structure in proteins by the DNN

We have previously successfully trained the FID-Net (*31*) architecture to virtually decouple and enhance the resolution of ^13^C-^1^H correlation spectra reporting on the methyl groups of large proteins (*35*). An initial attempt to use the same strategy for the aromatic region of ^13^C-^1^H correlation spectra of medium-to-large proteins was not satisfactory in our hands. We believe the reason for this is that the aromatic region of ^13^C-^1^H correlation spectra contains cross-peaks with different multiplet structures in the ^13^C dimension, whereas the methyl region essentially only contains doublets with a near-uniform splitting of about ~35 Hz. In the aromatic region, singlets are observed for histidine ^13^C^ε1^, doublets for tryptophan ^13^C^δ1^, and triplets for tyrosine and phenylalanine ^13^C^δ^ and ^13^C^ε^, respectively. Hence, the DNN (or a human) cannot differentiate between two singlets with the same ^1^H chemical shifts separated by ~55 to ~72 Hz from a doublet, making it nearly impossible to train the DNN to perform a robust transformation between coupled and uncoupled spectra. Similarly, two doublets with the same ^1^H chemical shifts, ^1^*J*_CC_ couplings and chemical shifts differing by ^1^*J*_CC_ can be mistaken for a triplet. To facilitate a robust transformation by the DNN for resolution enhancement, we decided to take several steps. The first step was to design an NMR experiment that provides unique information about the multiplet structure of the cross-peaks so that the trained DNN can uniquely distinguish the multiplet structure of the cross-peak that it is transforming into a singlet. The DNN should then be able to avoid converting a doublet into two singlets or a triplet into two singlets.

**Fig. 1.**
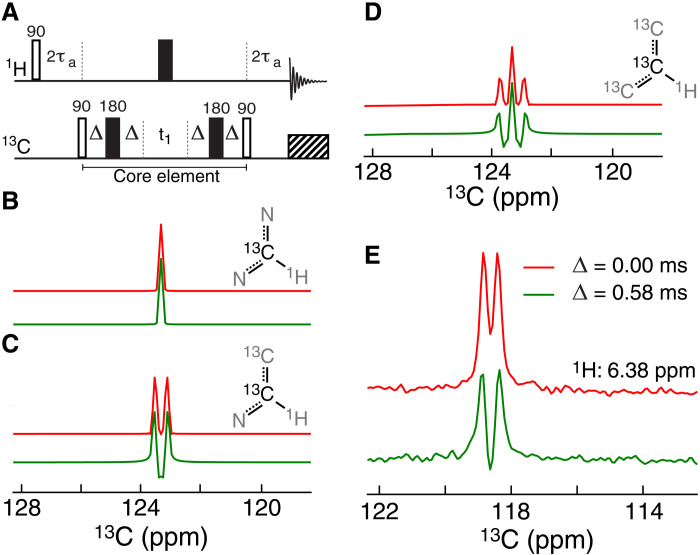
Encoding of unique features in ^13^C NMR spectra. (**A**) A simple depiction of the ^13^C-^1^H HMQC pulse sequence and the core element of the pulse sequence that allows for evolution of the scalar couplings and thus encodes unique features of the multiplet structure. The delays are τ_a_ = 1.4 ms and Δ as described below. The total coupling evolution, τ_coup_, is 4Δ. The chemical shift evolution time in the ^13^C dimension is denoted *t*_1_. Simulated 1D spectra showing the expected signals for a singlet (**B**), doublet (**C**), and a triplet (**D**) when the scalar couplings have been evolved for 0 ms (red; Δ = 0.0 ms) or 2.3 ms (green; Δ = 0.58 ms). ppm, parts per million. ^1^*J*_CC_ was set to 70 Hz, while the transverse relaxation rate was set 5 s^−1^. (**E**) 1D ^13^C slices of a ^13^C,^1^H correlation spectrum on L99A-T4L recorded at a temperature of 298 K and at a static magnetic field of 16.4 T. The slices are shown for the cross-peak arising from H31 ^13^C^δ2^-^1^H^δ2^ for Δ of 0.0 ms (red) and 0.58 ms (green).

Historically, NMR multiplet types have been distinguished using the distortionless enhancement by polarization transfer experiment, where scalar coupling evolutions are used to modulate the intensities of ^13^CH_3_, ^13^CH_2_, and ^13^CH groups differently. Building on a similar concept, the multiplet structure of the aromatic ^13^C-^1^H cross-peaks can be discerned by comparing two spectra: one corresponding to a normal ^13^C-^1^H HSQC spectrum and a second one in which the ^13^C-^13^C couplings have evolved for a small amount of time, τ_coup_ = 4Δ = 2.3 ms (~1/6 ^1^*J*_CC_) (Fig. 1A), in the indirect (^13^C) dimension. During the coupling evolution, magnetization arising from a singlet will not evolve, while the two lines of the doublet will evolve with frequencies corresponding to ±*J*_CC_/2 and the time evolution of the two lines can be succinctly represented as {exp(−*i* π *J*_CC_ τ_coup_), exp(*i* π *J*_CC_ τ_coup_)}. Along similar lines, the two outer lines of a triplet will evolve with frequencies corresponding to ±*J*_CC_, while the inner line, that is of twice the height, will not evolve and its phase can be represented as {exp(−2*i* π *J*_CC_ τ_coup_), 2, exp(2*i* π *J*_CC_ τ_coup_)}. Ignoring the effects of relaxation, spectra recorded with τ_coup_ = 0 and 2.3 ms will be indistinguishable from one another for a singlet. On the other hand, spectra recorded with τ_coup_ = 2.3 ms from doublet and triplet sites will contain a combination of absorptive and dispersive lineshapes, while the τ_coup_ = 0 ms spectra only contain absorptive lineshapes. Ideal spectra calculated for the pair of experiments [red τ_coup_ = 0 ms (red) and τ_coup_ = 2.3 ms (green)] are shown in Fig. 1B for a singlet, in Fig. 1C for a doublet, and in Fig. 1D for a triplet. Figure 1E shows a 1D ^13^C slice extracted from ^1^H-^13^C datasets recorded on L99A-T4L using the complementary pair of experiments described. The slice originates from the ^13^C^δ2^ site of H31, where the spectrum recorded with τ_coup_ = 0.0 ms is in red and the one recorded with τ_coup_ = 2.3 ms is shown in green. The multiplet pattern arising from the regular spectra (red) in Fig. 1E can arise either from two singlets or a doublet, but the spectrum recorded with τ_coup_ = 2.3 ms (green) that contains a combination of absorptive and dispersive lineshapes shows that it does not originate from two singlets (Fig. 1, B versus E) but from a doublet (Fig. 1, C versus E). Along similar lines, overlapping doublets can be distinguished from a triplet because the two components of the doublet evolve with frequencies of ±*J*_CC_/2 during the τ_coup_ = 2.3 ms delay, while the components of the triplet evolve with a different set of frequencies namely 0, ±^1^*J*_CC_ once again leading to different lineshapes in the spectra recorded with τ_coup_ = 2.3 ms. For an experimental example, see the application below to the A39F-FF domain. To summarize, the unique features, or pattern, generated by recording the second spectrum that incorporates evolution due to the ^13^C-^13^C coupling allows the DNN to identify the correct spin system.

### Training and assessing the performance of the FID-Net-2 DNN

To improve the spectral reconstruction from the two complementary datasets described above, we made several key changes to the FID-Net architecture that we have devised previously. We name this architecture FID-Net-2. The main difference between the original FID-Net and the FID-Net-2 architecture is that two complete 2D planes are processed within the architecture, as opposed to a sliding window of 1D spectra (fig. S1). Furthermore, FID-Net-2 outputs two sets of tensors (spectra), one output corresponding to the desired virtually decoupled and resolution-enhanced ^1^H,^13^C correlation spectrum, *I*(ϖ_H_,ϖ_C_), and a second tensor describing the uncertainty of the intensity for each point in the enhanced spectrum, σ(ϖ_H_,ϖ_C_). The architecture is described in detail in fig. S1. Training a DNN such as FID-Net-2 requires a large amount of training data. For FID-Net-2, the training data consist of the complementary HSQC datasets with (2.3 ms) and without evolution due to ^1^*J*_CC_ couplings and a target high-resolution HSQC spectrum free of splittings in the ^13^C dimension. In addition, passive couplings, small phase errors, Gaussian noise, roofing effects arising from deviations from the weak-coupling limit, and a residual solvent signal were all included in the training data, to make these as realistic as possible. Full details are given in Material and Methods and table S1. FID-Net-2 is then trained so that it learns to virtually decouple the desired high-resolution ^13^C-^1^H correlation map from the complementary HSQC datasets. The desired target high-resolution HSQC spectrum free of splittings in the ^13^C dimension cannot be experimentally obtained from a uniformly ^13^C-enriched sample and moreover would be infeasible to obtain for all the proteins required for training even if experimentally accessible. However, it is now established via several of our studies that DNNs for transforming experimental NMR spectra can be trained on synthetic data. The FID-Net-2 model was trained on approximately 30 × 10^6^ sets of synthetically generated spectra.

The loss function developed for training FID-Net-2 includes three parts, ${\text{Loss}}_{1}$, ${\text{Loss}}_{2}$, and ${\text{Loss}}_{3}$. ${\text{Loss}}_{1}$ corresponds to the traditional mean square error (MSE) between the target and predicted intensities. ${\text{Loss}}_{2}$ was designed to ensure a Gaussian distribution of the predicted uncertainties, and ${\text{Loss}}_{3}$ was designed to ensure that the uncertainties predicted agree with the root mean square deviation (RMSD) between the target and predicted spectra. See Materials and Methods for a detailed description of the training procedure. Last, it should be noted that FID-Net-2 can reconstruct high-resolution ^1^H-^13^C correlation maps from complementary HMQC or HSQC datasets because the same ^13^C chemical shift and the ^1^*J*_CC_ terms of the Hamiltonian are active during the *t*_1_ evolution period (^13^C dimension) in both of these experiments.

**Fig. 2.**
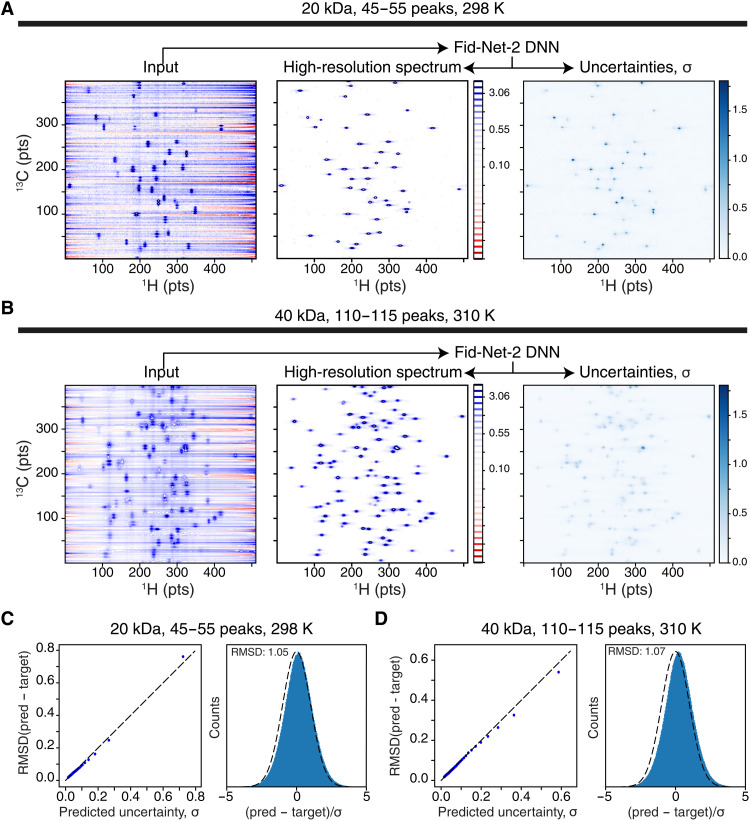
Transformation of synthetic spectra. (**A**) Transformation with FID-Net-2 of randomly generated synthetic data corresponding to a 20-kDa protein (298 K and 700 MHz). The transverse relaxation rates in the ^13^C and ^1^H dimensions were chosen from a random distribution with mean of 45 s^−1^ and SD of 20 s^−1^. Other parameters match those in table S1. (**B**) Transformation with FID-Net-2 of randomly generated synthetic data corresponding to a 40-kDa protein (310 K and 700 MHz). The transverse relaxation rates in the ^13^C and ^1^H dimensions were chosen from a random distribution with mean of 95 s^−1^ and SD of 20 s^−1^. Other parameters match those in table S1. (**C** and **D**) Assessment of the predicted error, where to the left is the χ_i_ versus RSMD and to the right is a histogram of the calculated χ_i_ = (pred*_i_* − target*_i_*)/σ*_i_*, showed a normal distribution with mean of nearly 0 and SD of nearly 1. The plots in (C) and (D) are calculated over 10 random spectra, each with a ${\text{Loss}}_{1}$ between 6.0 × 10^−3^ and 7.0 × 10^−3^, meaning that these data are representing data among the worst 40%.

We initially assessed the performance of the trained FID-Net-2 model on sets of synthetic data, where the advantage is that the ground truth is known. A summary of this assessment is shown in Fig. 2. Figure 2A shows a representative example where FID-Net-2 is applied to a spectrum expected from an approximately 20-kDa protein at 298 K. For such a case, we expect about 50 cross-peaks and transverse relaxation rates of about 45 ± 20 s^−1^ in both the ^13^C and ^1^H dimensions. In contrast to other DNN transformations of NMR data, FID-Net-2 transforms the input and produces two outputs, that is, the desired correlation spectrum (middle) and the uncertainty associated with the transformation (right). Note that the input consists of two 2D planes, whereas only one is shown in Fig. 2A.

First, it is seen that FID-Net-2 can eliminate the strong solvent signal to produce well-resolved spectra consisting of singlet cross-peaks. Of note is that the trained FID-Net-2 indeed produces point-by-point uncertainties, $\sigma_{i}$, that match what is expected, as judged from a Gaussian distribution of $\chi_{i}=\left(\text{targe}t_{i}-\text{predic}{\text{ted}}_{i}\right)/\sigma_{i}$, and predicted $\sigma_{i}$ that match the RMSD obtained from differences between predicted and target spectra (Fig. 2C). Figure 2B shows an application of FID-Net-2 to a simulated spectrum of a larger protein with a molecular mass of about 40 kDa. For such a protein, one expects about 110 cross-peaks in the aromatic region and transverse relaxation rates of about 95 ± 20 s^−1^. Again, the transformation of the input produces a clean well-resolved spectrum with predicted uncertainties that follow the desired criteria (Fig. 2D). Effectively, Fig. 2 (C and D) shows that the implementation of ${\text{Loss}}_{2}$ and ${\text{Loss}}_{3}$ was successful. With the accurate prediction of the uncertainties (Fig. 2, C and D), it also makes it easy for a user to judge when the reconstruction has been successful, for example, if a cross-peak in the spectrum has an intensity larger than the predicted uncertainty, then the position and intensity of the cross-peak can be trusted. However, if a small cross-peak observed in the reconstructed spectrum has an intensity that is comparable to the predicted uncertainty, then it is possible that the observed cross-peak is merely an artifact.

One could argue that real experimental spectra potentially contain features, or artifacts, that have not been included in the training data, or that there is the potential that a future user will obtain data that contains artifacts that have not been included in the simulation data. Thus, we have not aimed to include every possible artifact that a future user might encounter in the training data, but instead show that the trained FID-Net-2 model is robust when transforming data that contains artifact not included in the training set. To test the robustness of FID-Net-2, and in particular its ability to produce reliable error estimates, we produced synthetic data where the common artifact of *t*_1_ noise encountered in NMR spectroscopy was included (fig. S2). Although *t*_1_ noise was not included in the training data in any way, FID-Net-2 reconstructed the desired spectrum from the input data and more importantly predicted uncertainties that are only slightly underestimated from the expected ones (fig. S2). It is important to stress that it is not expected for FID-Net-2 to provide excellent reconstructions of spectra that contain *t*_1_ noise, or spectra that contain any other effects that have not been included in the training. However, it is important that FID-Net-2 provides reliable uncertainties for these out-of-distribution reconstructions. Thus, although this is not a comprehensive analysis of all possible artifacts, one can expect that, when situations that have not been included during training are encountered, FID-Net-2 will report larger errors that agree with the uncertainty of the predicted spectrum.

### FID-Net-2 reconstructs high-resolution aromatic ^13^C-^1^H correlation maps from experimental data

**Fig. 3.**
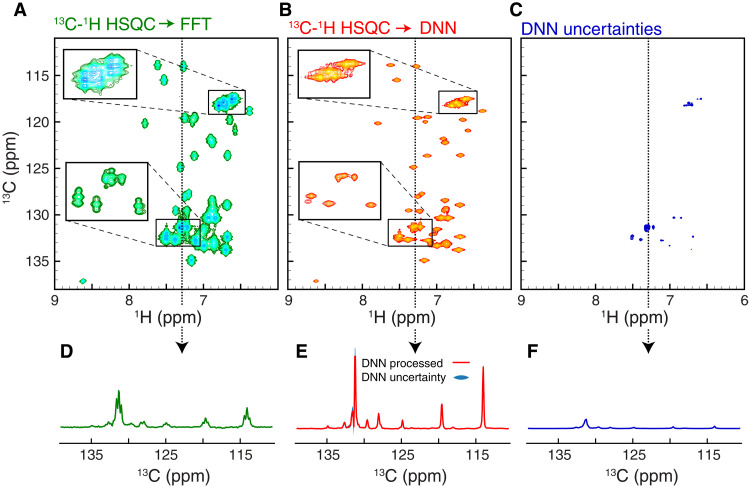
Transformation of experimental spectra of L99A-T4L. (**A**) ^13^C-^1^H HSQC spectrum reporting on the aromatic region of the 18-kDa L99A-T4L (298 K and 700 MHz). Correlations with different coupling multiplicity are clearly visible which leads to severe overlap in this medium size protein. (**B**) The high-resolution ^13^C-^1^H map reconstructed by FID-Net-2 from two ^13^C-^1^H HSQC spectra, recorded with τ_coup_ = 0.0 and 2.3 ms does not contain the multiplets seen in (A) leading to considerably lower overlap. (**C**) The uncertainty in the intensities of the reconstructed spectrum (B) predicted by FID-Net-2. (**D** to **F**) 1D representative slices of the spectra in (A), (B), and (C), respectively.

The spectral reconstructions and evaluations using synthetic data shown above are important to assess the limitations of the trained FID-Net-2 model. However, it is by applying FID-Net-2 to real experimental data that we will truly understand its capabilities. Initially, we recorded aromatic 2D ^13^C-^1^H HSQC correlation spectra of the 18 kDa, L99A mutant of lysozyme from the phage T4 (*36*) (L99A-T4L) at 16.4 T (700 MHz) (fig. S3). Apart from being relatively large compared to other proteins whose aromatic residues have been examined using NMR, L99A-T4L also exhibits conformational exchange that results in differential line-broadening, further testing the ability of FID-Net-2 to reconstruct high-resolution spectra from coupled spectra. As expected, using a traditional Fourier transform to process the ^13^C-^1^H HSQC data results in ^13^C-^1^H correlation maps with multiplets in the ^13^C dimension that show substantial overlap (Fig. 3A). In contrast, when the complementary pair (with and without the coupling delays) of ^13^C-^1^H HSQC datasets is processed using the FID-Net-2 model, a well-resolved spectrum of high quality is obtained with the expected number of peaks at their expected frequencies, which represents the accuracy of the FID-Net-2. Furthermore, the produced uncertainties are clearly not uniformly distributed over the spectrum as is the case for thermal noise processed with a linear Fourier transformation. It is well known that DNNs produce mappings that are highly nonlinear and one cannot therefore simply assess the performance, or accuracy, from the RMSD of a transformed spectrum in an area without cross-peaks, which is custom for standard processed spectra. The produced uncertainties in Fig. 3C clearly show that areas of the spectrum with highly overlapped peaks, which are typically challenging to analyze, result in substantial larger predicted uncertainties. The aromatic ^13^C-^1^H correlation maps reconstructed by FID-Net-2 from datasets with differing coupling delays are both better resolved and contain more signal compared to CT HSQC spectra (fig. S4). Specifically, a majority of peaks are missing in the CT HSQC spectra recorded with CT delay of 30.4 ms, even for the 18-kDa L99A-T4L protein. Although the signal-to-noise ratios of peaks in the CT HSQC spectrum recorded with a delay of 15.2 ms (fig. S4, D to F) are comparable to those in the FID-Net-2–transformed spectrum, the resolution is substantially less.

Having evaluated the trained FID-Net-2 model on synthetic data, including synthetic data with *t*_1_ noise, as well as on good-quality experimental data, we sought to further assess how the trained model behaves when the data contain artifacts that are not included in the training data. We did so experimentally by deliberately mis-setting the *Z*_1_ and *Z*_2_ shims of the NMR spectrometer to create an inhomogeneous field and thus create lineshapes that deviate markedly from the Lorentzian lineshapes used for training (fig. S5). For L99A-T4L, we recorded ^13^C-^1^H HSQC correlation spectra with optimal shimming and with nonoptimal shimming and subsequently compared peak intensities and peak positions, in line with the NUScon criteria (*37*). Excellent correlations are obtained both for peak positions and intensities (fig. S5), showing that FID-Net-2 can robustly reconstruct spectra from experimental data recorded under suboptimal conditions.

### Applications to larger proteins: FID-Net-2 reconstructs the high-resolution aromatic ^13^C-^1^H correlation map of 40-kDa *Escherichia coli* MBP

**Fig. 4.**
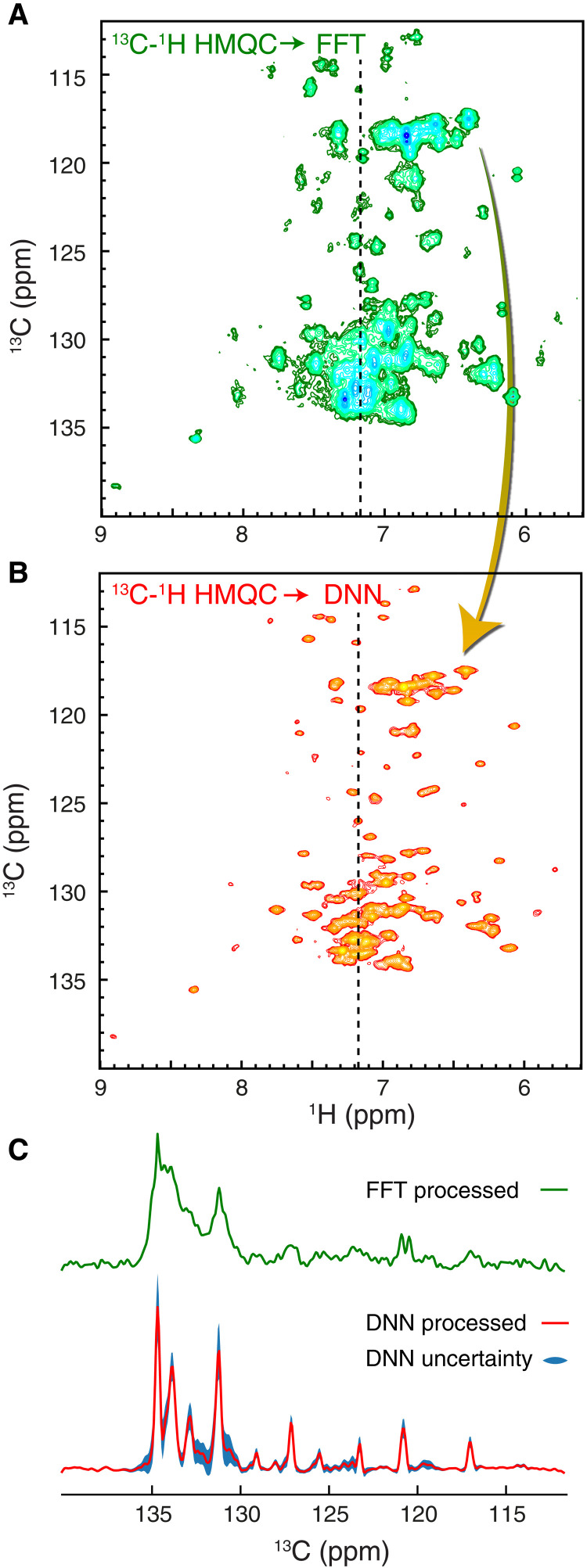
Transformation of experimental spectra of the 40-kDa MBP. (**A**) ^13^C-^1^H HMQC spectrum reporting on the aromatic region of the 40-kDa MBP, recorded at 310 K and at 700 MHz. Substantial overlap is observed with few resolved cross-peaks. (**B**) Processing with the FID-Net-2 model of two ^13^C-^1^H HMQC spectra, recorded with τ_coup_ = 0.0 and 2.3 ms. Many well-defined cross-peaks are observed, and the overlap is substantially less than in (A). (**C**) A 1D slice of the input ^13^C-^1^H HMQC spectrum is compared with the corresponding 1D slice of the output from FID-Net-2. The uncertainties predicted by the DNN model are shown as a blue filled area.

Recording high-resolution aromatic ^13^C-^1^H correlation maps for large proteins remains a challenge due to the short ^13^C transverse relaxation time that makes CT HSQC spectra very insensitive. The HMQC spectrum recorded on 40-kDa *E*. *coli* MBP in complex with β-cyclodextrin (MBP) at 310 K contains few resolved correlations (Fig. 4A), and a large number of correlations are severely overlapped because of *^1^J*_CC_ splittings in the indirect dimension. The ^13^C-^1^H correlation map reconstructed by FID-Net-2 however is much better resolved, once again demonstrating the efficacy of FID-Net-2 at reconstructing high-resolution aromatic ^13^C-^1^H correlation maps. We have chosen to use HMQC rather than HSQC type datasets as they are about 10% more sensitive (see fig. S6). The NOESY-based strategy described below can, in principle, be used for the assignment of the correlations in Fig. 4B, but this is beyond the scope of this work.

Using the 40-kDa MBP protein, with substantial peak overlap, we further assessed the FID-Net-2 mapping and the estimation of uncertainties. In summary, we recorded two sets of spectra, one with low signal-to-noise ratio (8 scans) and one set with high signal-to-noise ratio (128 scans). Since these spectra were recorded on the same sample using the same NMR spectrometer (700 MHz and 310 K), one expects that the signal intensities are proportional and that any deviations are captured by the uncertainties predicted by the trained FID-Net-2 model. Figure S7 shows an excellent correlation between the two transformed datasets, and it also shows that the deviations are well captured by the predicted uncertainties, thus providing further evidence that the trained FID-Net-2 model transforms the data accurately, even noisy data, and also produces quantitative uncertainties.

### Exploiting FID-Net-2 to obtain aromatic ^1^H-^13^C assignments from NOESY experiments

**Fig. 5.**
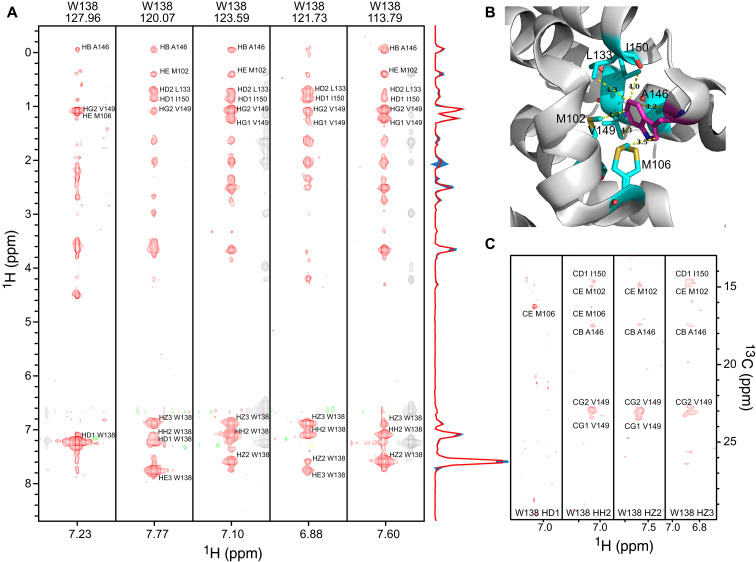
Aromatic ^1^H-^13^C assignments from NOESY spectra reconstructed using FID-Net-2. (**A**) Strips from the ^1^H-^1^H planes of the 3D ^1^H-^13^C_Aromatic_-^1^H_Aromatic_ NOESY spectrum of L99A-T4 lysozyme (25°C and 700 MHz) used for the assignment of Trp^138^. (**B**) The residue Trp^138^ is highlighted as magenta sticks on a cartoon representation of the T4 Lysozyme structure (Protein Data Bank ID: 3dmv) (*50*). The residues in close proximity to Trp^138^ are shown in cyan sticks and their distances from the aromatic side chain of Trp^138^ are also shown in the figure. (**C**) Strips from the ^1^H-^13^C planes of the 3D ^13^C_Methyl_-^13^C_Aromatic_-^1^H_Aromatic_ NOESY spectrum of L99A-T4 lysozyme (25°C and 700 MHz) focusing on Trp^138^. The structure of the protein was used to identify aromatic and methyl protons that are close to one another, following which the complementary pair of 3D NOESY spectra that contain cross-peaks between aromatic and methyl protons that are proximal to one another was used to assign the aromatic ^1^H and ^13^C resonances. FID-Net-2 was used to process the ^13^C_Aromatic_-^1^H_Aromatic_ dimensions.

Obtaining aromatic ^1^H and ^13^C assignments in medium-sized proteins is challenging because HSQC-NOESY–type spectra have poor resolution in the aromatic ^13^C dimension due to ^1^*J*_CC_ couplings, while the CT-HSQC-NOESY spectra suffer from poor signal-to-noise ratio due to the short transverse relaxation times of aromatic ^13^C nuclei. FID-Net-2 provides a ready solution to the problem. To assign the chemical shifts of the aromatic ^13^C-^1^H spectrum of L99A-T4L, we recorded ^13^C_Methyl_-^13^C_Aromatic_-^1^H_Aromatic_ and ^1^H-^13^C_Aromatic_-^1^H_Aromatic_ 3D NOESY spectra (fig. S8) and processed these with FID-Net-2 in the ^13^C_Aromatic_-^1^H_Aromatic_ dimensions. A summary of these spectra and the chemical shift assignment procedure that uses ^13^C,^1^H methyl assignments are shown in Fig. 5. Figure 5A highlights how the uncertainties in intensity provided by FID-Net-2 aid in analyzing the NOESY spectra. Cross-peaks with uncertainties that are as large as the signal intensities should be very carefully assessed, whereas cross-peaks (even weak ones) with small uncertainties can be confidently interpreted. On the basis of a previous ^13^C,^1^H methyl assignment (*38*), these two spectra were sufficient to assign the correlations seen in the high-resolution aromatic ^13^C-^1^H correlation map (fig. S9).

### Quantitative characterization of protein dynamics using FID-Net-2

**Fig. 6.**
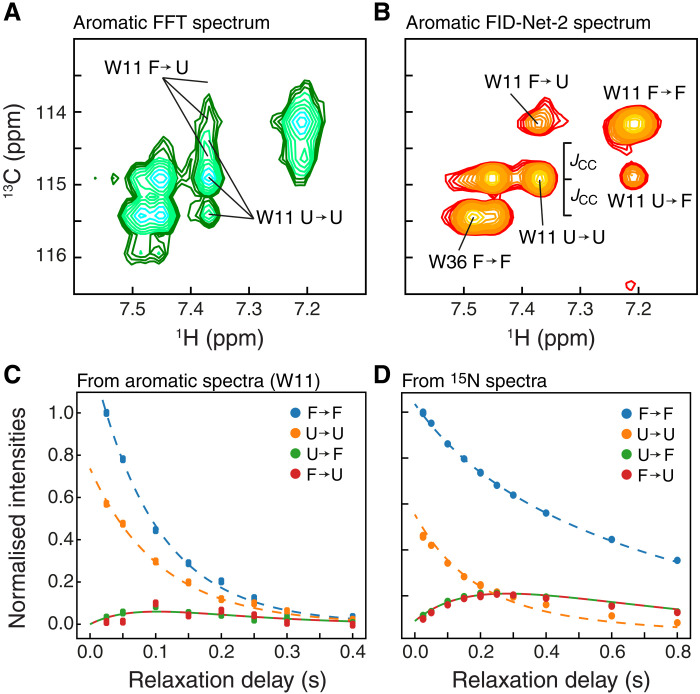
Transformations with FID-Net-2 are quantitative. Regular ^13^C-^1^H correlation map (**A**) and the FID-Net-2 reconstructed ^13^C-^1^H correlation map from a ZZ exchange (*T*_EX_ = 150 ms) experiment (**B**) reporting on the aromatic region of the 7-kDa A39G mutant FF domain in the presence of 1 M urea (275 K and 600 MHz). Both spectra contain peaks arising from the folded (F) and the unfolded (U) state of the protein. The regular (FFT) spectrum (A) is severely overlapped while the FID-Net-2 reconstructed spectrum (B) is much better resolved allowing one to identify both diagonal (F → F and U → U) and exchange cross-peaks (F → U and U → F) arising from the ^13^C^ζ2^-^1^H^ζ2^ site in W11. (**C**) Intensities extracted from (B) for various *T*_EX_ delays were analyzed using the standard Bloch-McConnnell formalism (*51*) to obtain the exchange parameters. The dashed lines are drawn using the best fit parameters (*k*_ex_ = 3.39 ± 0.32 s^−1^ and *p*_U_ = 36.3 ± 1.7%). (**D**) Intensities extracted from a ^15^N ZZ exchange experiment on the same sample, for diagonal (F → F and U → U) and exchange peaks (F → U and U → F). The dashed lines are drawn using the best fit exchange parameters (*k*_ex_ = 4.08 ± 0.17 s^−1^ and *p*_U_ = 38.7 ± 0.8%).

Previous DNNs devised to transform NMR spectra were not quantitative with respect to the intensities of cross-peaks (*35*) and were not useful to study chemical exchange, characterize binding, or other studies where accurate peak intensities are necessary. FID-Net-2 was trained to be quantitative in this regard, although it needs to be kept in mind that peaks are being sharpened in the ^13^C dimension, and analysis that involves classical direct lineshape analysis should be avoided. To exploit the quantitative aspect of FID-Net-2, we recorded longitudinal exchange (*39*) spectra (EXSY/ZZ exchange) on the A39G-FF domain (Fig. 6). The EXSY/ZZ exchange experiment can be used to quantify slow chemical or conformational exchange processes, when the exchange rate is between ca. 0.1 and 10 s^−1^. In these experiments, the exchange of longitudinal magnetization between the major and minor peaks is observed as a function of time. It is thus required that both cross-peaks from both the major and the minor conformations are observed. The aromatic ^13^C,^1^H chemical shift assignment of A39G-FF was obtained using the 3D NOESY spectra described above (fig. S10). A39G-FF exchanges slowly between the folded state and the unfolded state (*40*) and the addition of a small amount of urea (1 M) increases the unfolded state population giving rise to two sets of peaks in NMR spectra. As seen in Fig. 6B, the FID-Net-2–transformed ^13^C-^1^H correlation map clearly shows the two sets of cross-peaks reporting on the exchange between the folded and unfolded states of A39G-FF. A least-squares analysis of the data provided the exchange rate (*k*_ex_) and the population of the unfolded species (*p*_U_). To assess the quality of the data, we also recorded ^15^N,^1^H ZZ exchange spectra and obtained an exchange rate and a population (*k*_ex_ = 4.1 ± 0.2 s^−1^ and *p*_U_ = 38.7 ± 0.8%) in agreement with those obtained from the FID-Net-2–transformed spectra (*k*_ex_ = 3.4 ± 0.3 s^−1^ and *p*_U_ = 36.3 ± 1.7%), thus experimentally demonstrating that spectra transformed with FID-Net-2 can be used for quantitative analyses. The slightly larger uncertainty of the aromatic FID-Net-2 data compared to the ^15^N data is mainly caused by the faster *R*_1_ relaxation of the coherence present in the aromatic experiment, 2C_z_H_z_, compared to that present in the ^15^N experiment, ^15^N_z_.

## DISCUSSION

Being able to characterize the regulation, interactions, and dynamics of medium and large proteins in solution is paramount for understanding their molecular functions. To that end, it is imperative to have tools to characterize aromatic side chains in proteins that are critical reporters of function because these sites are often located in interaction hot spots, involved with substrate binding, regulation, and catalysis.

Specific isotopic labeling (*23*–*25*, *41*, *42*) has been one of the only means to characterize aromatic residues in medium-sized proteins. However, these labeling schemes limit the number of probes available and require the use of specific precursors that often lead to reduced protein yield of the samples. Here, we presented an attractive alternate method to characterize functional aromatic residues in medium-sized proteins, wherein a pair of complementary ^1^H-^13^C datasets recorded using a uniformly ^13^C-isotopically enriched protein sample are processed with the FID-Net-2 model to obtain the desired high-resolution aromatic ^13^C-^1^H correlation map. It is important to note that this methodology, based on processing with a DNN, offers simultaneous access to all the ^13^C-^1^H spin pairs in all the aromatic side chains in the protein and does not require specifically labeled samples. The FID-Net-2 network architecture provides a different way to transform NMR data using DNNs, because it not only produces resolution-enhanced spectra but also provides a good estimate of the uncertainty in the intensities of these spectra. We have exploited these abilities of FID-Net-2 by obtaining chemical shift assignments (L99A-T4L) and characterizing chemical exchange (A39G-FF). For both of these applications, it was necessary to have accurate estimates of the uncertainties in the intensities. For the analysis of NOESY data (L99A-T4L), the uncertainties are used to distinguish artifacts from real cross-peaks. On the other hand, while studying exchange (A39G-FF) the uncertainties aid in carrying out a robust least-squares analysis. We believe that this methodology will allow for a general and easy characterization of functional aromatic side chains in medium-sized proteins.

Two major developments contribute to the success of FID-Net-2: (i) the design of new NMR experiments with the sole goal of aiding the DNN and (ii) training the DNN to estimate uncertainties of the transformed spectra. Datasets with τ_coup_ set to 2.3 ms are recorded solely to provide unique features for the DNN to analyze. Because of ^1^*J*_CC_ evolutions during τ_coup_, spectra obtained from such datasets will contain dispersive components in the ^13^C dimension making them unappealing to a human NMR spectroscopist, but nonetheless useful to the DNN that uses the information present in such datasets to reconstruct high-resolution ^1^H-^13^C correlation maps. The uncertainties estimated by FID-Net-2 are crucial to both applications presented here. Knowledge of the uncertainties was critical for both identifying “valid” cross-peaks in the NOESY spectra for the purposes of assignment and for obtaining kinetic parameters from the variation of cross-peak intensities as a function of mixing time. As with other convolutional neural networks, it is likely that the trained FID-Net-2 model presented in this study can be retrained to transform other types of spectra.

It is now clear that processing and transforming NMR spectra with DNNs is a powerful tool. However, we believe that to truly exploit the potential of DNNs in NMR, it is not enough to just devise new DNNs that transform existing experimental data, but to devise new experiments specifically for the DNNs to exploit as we have done here. Concomitantly developing DNNs and experimental methods will in the future to come allow for advanced insights, in AI-assisted NMR spectroscopy and likely also in other related scientific fields.

## MATERIALS AND METHODS

### The FID-Net-2 architecture

We aimed to develop a DNN to map ^13^C-^1^H correlation NMR spectra reporting on the aromatic region of uniformly ^13^C-labeled proteins into spectra of high resolution. Standard ^13^C-^1^H spectra of uniformly labeled proteins are affected by one-bond ^13^C-^13^C homonuclear scalar couplings, line broadenings, and residual solvent signals. The developed DNN will therefore need to (i) virtually decouple the multiplet structures arising from the homonuclear couplings, (ii) generally enhance the resolution, and (iii) remove solvent signals. Last, (iv) we also require that the DNN is able to predict the accuracy with which it does the mapping, which means that the DNN provides point-by-point uncertainties σ(ϖ_1H_,ϖ_13C_), of the predicted output *I*(ϖ_1H_,ϖ_13C_). As noted in the main text and Fig. 1, two input spectra are required for this transformation to be robust. It should be noted that the mapping performed by the developed DNN will not increase the information in the provided data but will combine the information in the two input spectra and generate a spectrum that is of high resolution and easily interpretable by the end-user spectroscopist.

To achieve the above requirements for the DNN, the previous FID-Net architecture (*31*) was substantially altered in several ways, including, (i) full 2D planes are transformed as opposed to using a sliding window, (ii) both the ^13^C and ^1^H dimensions are processed within the same architecture, (iii) a refinement step in the frequency domain was included in the end, and (iv) uncertainties are also predicted. Of note is that the last layer of FID-Net-2 produces a tensor of size (512,400,2), where the first (512,400) plane is the ^1^H-^13^C resolution-enhanced spectrum and the second (512,400) plane is the confidences. A sigmoidal activation, 1/[exp(−*x*) + 1], is used to ensure that the confidences take values between 0 and 1. SDs are calculated from the confidence, conf, by

$$\sigma =\frac{1}{0.998\times \text{sigmoid}\left(\text{conf}\right)+0.001}-1$$

Last, the predicted spectrum and the predicted uncertainties, σ, are convolved with a sine-bell window function, with offset of 0.4π, before calculating the losses. The architecture is detailed in fig. S1.

### Synthetic spectra for training FID-Net-2

The FID-Net-2 DNN was trained exclusively on synthetic data, summarized in Fig. 2 and subsequently evaluated on synthetic data and experimentally acquired from protein samples. The resolution in the ^13^C dimension was enhanced both with virtual decoupling and by decreasing the effective transverse relaxation rate. When decreasing the effective transverse relaxation rate, care must be taken, so that the DNN does not generate artifacts from very broad features in the spectrum. We found that halving the effective relaxation rate worked well in the ^13^C dimension, that is, *R*_2,tar_ = 0.5 *R*_2,inp_, where the input rates, *R*_2,inp_, were randomly generated from a normal distribution with mean of 50 s^−1^ and SD of 20 s^−1^ and *R*_2,tar_ is the target transverse relaxation rate. The multiplet structures of the ^13^C-^13^C couplings in the input spectrum were simulated by generating two sets of coupling constants, *J*_1,C_ and *J*_2,C_, that were each drawn from a normal distribution with mean of 63 Hz and SD of 10 Hz. Subsequently, 20% of *J*_1,C_ and 20% of *J*_2,C_ were set to zero, which results in 64% triplet structures, 4% singlet structures, and 32% doublet structures. To simulate non-weak couplings, roofing effects were added by multiplying the FID in the ^13^C dimension by $\left\{\text{cos}\left(\pi J_{1,C}t_{1}\right)+\varrho_{1}\;i\;\text{sin}\left(\pi J_{1,C}t_{1}\right)\right\}\times \left\{\text{cos}\left(\pi J_{2,C}t_{1}\right)+\varrho_{2}\;i\;\text{sin}\left(\pi J_{2,C}t_{1}\right)\right\}$. Here, ρ_1_ and ρ_2_ are factors to include roofing effects and these are both drawn from a normal distribution with mean of 0 and SD of 0.08. Each spectrum contained between 40 and 200 cross-peaks, with chemical shifts uniformly distributed along the ^13^C dimension. The ^1^H chemical shifts were generated to increase the overlap of cross-peaks. First, initial ^1^H chemical shifts $\delta_{H}^{0}$ were drawn from a normal distribution with mean of 0 part per million and SD of SW/4. Subsequently, to increase the overlap, the final ^1^H chemical shifts were calculated using the following empirical equation, which also ensures that cross-peaks are not on the edge of the spectrum in the ^1^H dimension

$$\delta_{H}=0.2\times \text{SW}\times \text{tanh}\left({\left\{\frac{2\delta_{H}^{0}}{\text{SW}}\right\}}^{3}\right)+0.3\times \text{SW}\times \text{tanh}\left(\frac{2\delta_{H}^{0}}{\text{SW}}\right)$$

In the ^1^H dimension, the input simulated data included ^1^H-^1^H homonuclear couplings. Similar to the ^13^C dimension, two sets of coupling constants were generated *J*_1,H_ with an average of 8 Hz and a SD of 2 Hz and *J*_2,H_ with an average of 4 Hz and a SD of 2 Hz; 10% of *J*_1,H_ were set to zero and 50% of *J*_2,H_ were set to zero. Roofing effects, which are non-weak couplings, were simulated in the same way as for the ^13^C dimension. Solvent signals were simulated in the ^1^H frequency domain as

$$\text{solvent}\left(i\right)=10\times \text{int}\times \left\{\text{slp}\left(\frac{1}{x_{i}+0.1}-0.9\right)-\left(1-\text{slp}\right)x_{i}\right\}$$

where int is a random number drawn from the same normal distribution used to assign peak intensities, and slp is a random number between 0 and 1 (uniform). When the 1D ^1^H frequency-domain spectrum contains *N* points, then $x_{i}$ is 0/*N*, …, (*N* − 1)/*N*. Half of the solvent residuals were inverted in the ^1^H dimension, such that the DNN learned to deal with solvent signals from both the left and the right side of the spectrum. Last, the residual solvent signal generated in frequency domain was Fourier transformed to generate the solvent signal in the time domain, which was added to the synthetically generated random spectrum.

The FID-Net-2 model was trained on a diverse range of NMR parameters (table S1) and so can be used without need for further retraining and the approach can be used with standard ^1^H-^13^C HSQC or HMQC pulse sequences.

### Training the FID-Net-2 architecture with synthetic spectra

The FID-Net-2 model was trained on approximately 30 × 10^6^ sets of spectra, where one set consisted of a target 2D spectrum (target) and two input spectra, without coupling evolution, input_no-coup_, and with 2.3-ms coupling evolution in the ^13^C dimension, input_coup_. In brief, chemical shifts were randomly distributed in the ^13^C dimension, while more condensed in the ^1^H dimension to mimic increased overlap. For the input spectra, we also added random Gaussian noise and a solvent signal akin to a residual water signal. A maximum of 200 cross-peaks were generated. All training parameters are provided in table S1. The DNN model (fig. S1) was developed and trained using the TENSORFLOW 2.11 library (*43*) with the KERAS (*44*). As detailed in table S1, the model has been trained with the number of complex input points varying between 96 and 200 in the ^13^C dimension and between 128 and 256 in the ^1^H dimension. An initial step will zero-fill the time domains to 200 complex points in ^13^C and 256 in ^1^H, such that the input to the DNN is a tensor of constant size (2,400,512). Similarly for inference after the model has been trained.

As mentioned in the text specialized loss functions were used to train the network with the total Loss consisting of three parts, ${\text{Loss}}_{1}$, ${\text{Loss}}_{2}$, and ${\text{Loss}}_{3}$. ${\text{Loss}}_{1}$ corresponds to the traditional idea of minimizing the difference between the predicted output spectrum and the target spectrum, whereas ${\text{Loss}}_{2}$ is restraining a Gaussian distribution of the predicted errors, and ${\text{Loss}}_{3}$ is restraining the calculated uncertainties to match the RMSD between the predicted and target spectrum, over 200 linear bins. Specifically, the function for ${\text{Loss}}_{1}$, also referred to as MSE, is simply defined as

$$\text{Los}s_{1}=\frac{1}{N}\sum_{i}{\left(\text{targe}t_{i}-\text{predic}t_{i}\right)}^{2}$$ (1)

Where the sum is over all points in the spectrum and N is the total number of points in the 2D plane (400 × 512). The losses ${\text{Loss}}_{2}$ and ${\text{Loss}}_{3}$ were designed specifically for Fid-Net-2. For ${\text{Loss}}_{2}$, a value $\chi_{i}$ was first calculated as

$$\chi_{i}=\frac{\text{targe}t_{i}-\text{predic}t_{i}}{\sigma_{i}}$$ (2)

where $\sigma_{i}$ is the predicted error (output from FID-Net-2). Our goal was to have $\chi_{i}$ follow a standard Gaussian distribution with zero mean and SD of 1. To achieve this, the 1/2th, first, second, third, and 7/2th momenta of $\chi_{i}$ were restrained as follows

$$\text{Los}s_{2}=\sum_{m\in \left\{\frac{1}{2};1;2;3;\frac{7}{2}\right\}}{\left\{\left(\frac{1}{N}\sum_{i}\chi_{i}^{m}\right)-M_{m}\right\}}^{2}$$ (3)

where $M_{\frac{1}{2}}={\left(-\frac{1}{2}\right)}^{\frac{1}{4}}\Gamma \left(\frac{3}{4}\right)/\sqrt{\pi }$, $M_{1}=0$, $M_{2}=1$, $M_{3}=0$, $M_{7/2}=$ $2^{3/4}\;\left(1-i\right)\;\Gamma \left(\frac{9}{4}\right)/\sqrt{\pi }$, and Γ() is the gamma function.

For the calculation of ${\text{Loss}}_{3}$, the predicted errors, $\sigma_{i}$, were binned into 200 bins (linear), with the bins equally spaced between 0 and max($\sigma_{i}$). Within each of these 200 bins, the average of the $\sigma_{i}$ was calculated and restrained to be equal to the RMSD between the predicted points and the target points, for points corresponding to this bin. Specifically

$$\text{Los}s_{3}=\sum_{b\in \text{bins}}{\left\{\frac{1}{N_{b}}\sum_{i\in b}\sigma_{i}-\sqrt{\frac{1}{N_{b}}\sum_{i\in b}{\left(\text{targe}t_{i}-\text{predic}t_{i}\right)}^{2}}\right\}}^{2}$$ (4)

During initial training, only Loss_1_ was included, since uncertainties cannot be predicted until a reasonable prediction of the reconstructed spectrum can be achieved. When ${\text{Loss}}_{1}$ reached 0.03, ${\text{Loss}}_{2}$ and ${\text{Loss}}_{3}$ were slowly introduced as a scaling, specifically

$$\text{Scaling}=\text{sigmoid}\left\{-\text{exp}\left[100*\text{min}\left({\text{Loss}}_{1},0.03\right)\right]\right\}/0.268$$

Subsequently, ${\text{Loss}}_{\text{total}}=\left(2-\text{Scaling}\right)*{\text{Loss}}_{1}+\text{Scaling}*\left({\text{Loss}}_{2}+{\text{Loss}}_{3}\right)$. The model was trained to a total loss of 8.7 × 10^−3^ and ${\text{Loss}}_{1}$ of ca. 5 × 10^−3^. Below, the trained model is first assessed on synthetic data and subsequently we evaluate the model on a series of experimental data.

The ADAM (*45*) optimizer was used for training with a learning rate that changed throughout the training, β_1_ = 0.9, β_2_ = 0.98, and ε = 10^−9^. Mini-batching was used with four sets of spectra in each mini-batch and the weights saved for every 2000 batches. The learning rate, lr, was calculated as

$$\begin{array}{c} \text{lr}\left(\text{step}\right)=\frac{1}{2884}\text{min}\left(a_{1},a_{2}\right),\\ \text{where}\;a_{1}=\frac{1}{\sqrt{\text{step}}}\;\text{and}\;a_{2}=3.5\times 10^{-7}\text{step} \end{array}$$

The parameter, step, is a counter for batches used in training. Thus, after an initial warm-up period, the largest learning rate used is about 2.45 × 10^−6^, whereafter the learning rate decays. The FID-Net-2 architecture was trained on the NMRBox facility (*46*) using two Nvidia A100 GPUs.

### Assessment using synthetic data

Once trained, the performance of the trained FID-Net-2 model was initially evaluated on synthetically generated data, as shown in Fig. 2. Two independent assessments were made, one to represent a protein of about 20 kDa and one to represent a protein of about 40 kDa. Parameters used to generate the synthetic spectra were the same as those used for training, table S1, except that for the 20-kDa protein, only between 45 and 55 cross-peaks were generated with *R*_2_(^1^H) and *R*_2_(^13^C) both drawn from a normal distribution with mean of 45 s^−1^ and SD of 20 s^−1^. For the 40-kDa protein, 110 to 120 cross-peaks were generated with *R*_2_(^1^H) and *R*_2_(^13^C) both drawn from a normal distribution with mean of 95 s^−1^ and SD of 20 s^−1^.

### NMR samples

All three [U-^15^N^13^C] protein samples were prepared by over expressing the proteins in *E. coli* BL21(DE3) cells transformed with the appropriate plasmids and grown in M9 medium supplemented with ^15^NH_4_Cl (1 g/liter) and ^13^C-glucose (3 g/liter) as nitrogen and carbon sources, respectively. L99A-T4L (*38*), A39G-FF (*40*), and MBP (*47*) were all purified as described previously.

The L99A-T4L sample consisted of ~1 mM [U-^15^N^13^C] protein dissolved in 50 mM sodium phosphate, 25 mM NaCl, 2 mM EDTA, 2 mM NaN_3_, and ~99% D_2_O, pH 5.5 buffer. The A39G-FF samples consisted of ~1 mM [U-^15^N^13^C] protein dissolved in 50 mM sodium acetate, 100 mM NaCl, 2 mM EDTA, and 2% D_2_O, pH 5.7 buffer. The MBP samples consisted of ~0.5 mM [U-^15^N^13^C] protein dissolved in 20 mM sodium phosphate, 1 mM EDTA, 2 mM β-cyclodextrin, and 99% D_2_O, pH 6.5 buffer.

### NMR experiments

All NMR spectra of L99A-T4L, MBP, and A39G-FF were acquired on a 1 mM (L99A-T4L), a 0.5 mM (MBP), or a 0.5 mM (A39G-FF) uniformly [^13^C,^15^N]-labeled samples on a Bruker 700 MHz Avance III spectrometer equipped with Z-gradient triple-resonance TCI cryoprobe, unless otherwise specified.

#### 2D ^13^C-^1^H correlation spectra

All 2D HSQC and HMQC datasets of L99A-T4L (Fig. 3), MBP (Fig. 4), and A39G-FF used as input for FID-Net-2 were recorded using the pulse sequences described in fig. S3 (A and B), with τ_coup_ = 0.0 and 2.3 ms. The data were acquired with 768 and 200 (L99A-T4L and A39G-FF) or 768 and 128 (MBP) complex points in the ^1^H and ^13^C dimensions, respectively, with spectral widths of 10,000 and 5000 Hz. The datasets were recorded as pseudo-3D spectra. An interscan delay of 1 s was used. For the experiments below, the same spectral width and interscan delay were used, unless otherwise specified.

The CT experiments (fig. S4) of L99A-T4L were recorded using a standard Bruker pulse sequence (hsqcctetgpsp) with coherence-selection gradients. The data were acquired with 768 and 128 (30.4 ms CT) or 60 (15.2 ms CT) complex points in the ^1^H and ^13^C dimensions, respectively, at 278 K. HMQC-type spectra of L99A-T4L was used to assess the out-of-scope behavior of FID-Net-2 and with poor shimming (fig. S5). The data were recorded using the pulse sequences described in fig. S3B, with τ_coup_ = 0.0 ms and 2.3 ms, on a Bruker 600 MHz Avance HD spectrometer equipped with Z-gradient triple-resonance TXO cryoprobe. The data were acquired with 768 and 200 complex points in the ^1^H and ^13^C dimensions, respectively, with spectral widths of 9009 and 5000 Hz.

#### 3D NOESY spectra

The 3D ^1^H-^13^C-^1^H NOESY dataset of L99A-T4L (Fig. 5A) and A39G-FF used as input for FID-Net-2 were recorded using the pulse sequences described in fig. S3D. The data were acquired with 1024, 128, and 96 complex points in ^1^H, ^1^H_NOESY_, and ^13^C_Aro_ dimensions, respectively, with spectral widths of 14280 (^1^H), 5000 (^13^C), and 8000 Hz (^1^H_NOESY_). Four scans were collected per increment. The NOESY mixing time was 100 ms for L99A-T4L sample and 200 ms for the A39F-FF sample.

The 3D ^1^H-^13^C_Aro_-^13^C_Methyl_ datasets of L99A-T4L (Fig. 5C) and A39G-FF used as input for FID-Net-2 were recorded using the pulse sequences described in fig. S8. The data were acquired with 1024, 128, and 80 complex points in ^1^H, ^13^C_NOESY_, and ^13^C_Aro_ dimensions, respectively, with spectral widths of 14280 (^1^H), 5000 (^13^C_Aro_), and 5000 Hz (^1^C_Methyl_). Four scans were collected per increment. The NOESY mixing time was 120 ms for the L99A-T4L sample and 200 ms for the A39F-FF sample.

#### Longitudinal exchange (EXSY; ZZ exchange) spectra

The longitudinal aromatic ^13^C,^1^H exchange dataset and ^15^N,^1^H exchange dataset of A39G-FF (Fig. 6) were recorded on a 0.5 mM uniformly [^13^C,^15^N]-labeled samples using the pulse sequences described in fig. S3C, with τ_coup_ = 0.0 and 2.3 ms, on a Bruker 600 MHz Avance HD spectrometer equipped with Z-gradient triple-resonance TCI cryoprobe at 274 K. The longitudinal aromatic ^13^C,^1^H exchange data were acquired with 768 and 128 complex points in ^1^H and ^13^C_Aro_ dimensions, respectively, with spectral widths of 9009 (^1^H) and 5000 Hz (^13^C). Sixteen scans were collected per increment and the exchange delays of 25, 50, 100, 150, 200, 250, and 300 ms were used. The longitudinal aromatic ^15^N,^1^H exchange dataset of A39G-FF (Fig. 6) was acquired with 1536 and 128 complex points in ^1^H and ^15^N dimensions, respectively, with spectral widths of 10000 (^1^H) and 2136 Hz (^13^C). Eight scans were collected per increment and the exchange delays of 25 (duplicate), 50, 100, 150, 200 (duplicate), 250, 300, 400, 600, and 800 ms were used.

#### Data processing

All experimental NMR spectra were processed with NMRPIPE (*48*) or using the Python libraries NMRGLUE (*49*) and NUMPY.

## Supplementary Materials

### This PDF file includes:

Table S1 Figs. S1 to S10 References
