# Supplementary material for: A combined NMR and deep neural network approach for enhancing the spectral resolution of aromatic side chains in proteins
Source: Sci Adv. 2024 Dec 20;10(51):eadr2155. doi: 10.1126/sciadv.adr2155 (PMC11801238; doi:10.1126/sciadv.adr2155)
Supplement: Supplementary file 1 — Table S1 Figs. S1 to S10 References [file sciadv.adr2155_sm.pdf]

Science Advances
AAAS

Supplementary Materials for

A combined NMR and deep neural network approach for enhancing the spectral resolution of aromatic side chains in proteins

Vaibhav Kumar Shukla *et al.*

Corresponding author: D. Flemming Hansen, d.hansen@ucl.ac.uk

Sci. Adv. **10**, eadr2155 (2024)
DOI: 10.1126/sciadv.adr2155

This PDF file includes:

Table S1
Figs. S1 to S10
References

Supporting Tables:

Table S1. Parameters used for Training

**Parameter**	
Number of cross-peaks	40 – 200 (uniform distribution)
Intensity	$\mathcal{N}(1, 0.5)$[a)]
Noise	$\mathcal{N}(1.2, 0.12)$
^{1}H SW (Hz)	2000 – 5000
^{1}H number of complex points	128 – 256
$R_2(^1H)$ (s^{-1})	$\mid \mathcal{N}(50, 20) \mid$
Phase(^{1}H)	$\mathcal{N}(0, 10)$
J_{HH} (Hz)	$\mathcal{N}(8, 2)$ [10% set to zero]
J_{HH} (Hz)	$\mathcal{N}(4, 2)$ [50% set to zero]
^{13}C SW (Hz)	4000 – 6650
^{13}C Acquisition time (s)	0.030 – 0.050 (uniform distribution)
^{13}C number of complex points	96 – 200 (uniform distribution)
$R_2(^{13}C)$ (s^{-1})	$\mid \mathcal{N}(50, 20) \mid$
Phase(^{13}C)	$\mathcal{N}(0, 10)$
J_{CC} (Hz)	$\mathcal{N}(63, 10)$ [20% set to zero]
J_{CC} (Hz)	$\mathcal{N}(63, 10)$ [20% set to zero]

a) $\mathcal{N}(\mu, \sigma)$ is a normal distribution with mean μ and standard deviation σ.

Supporting Figures:

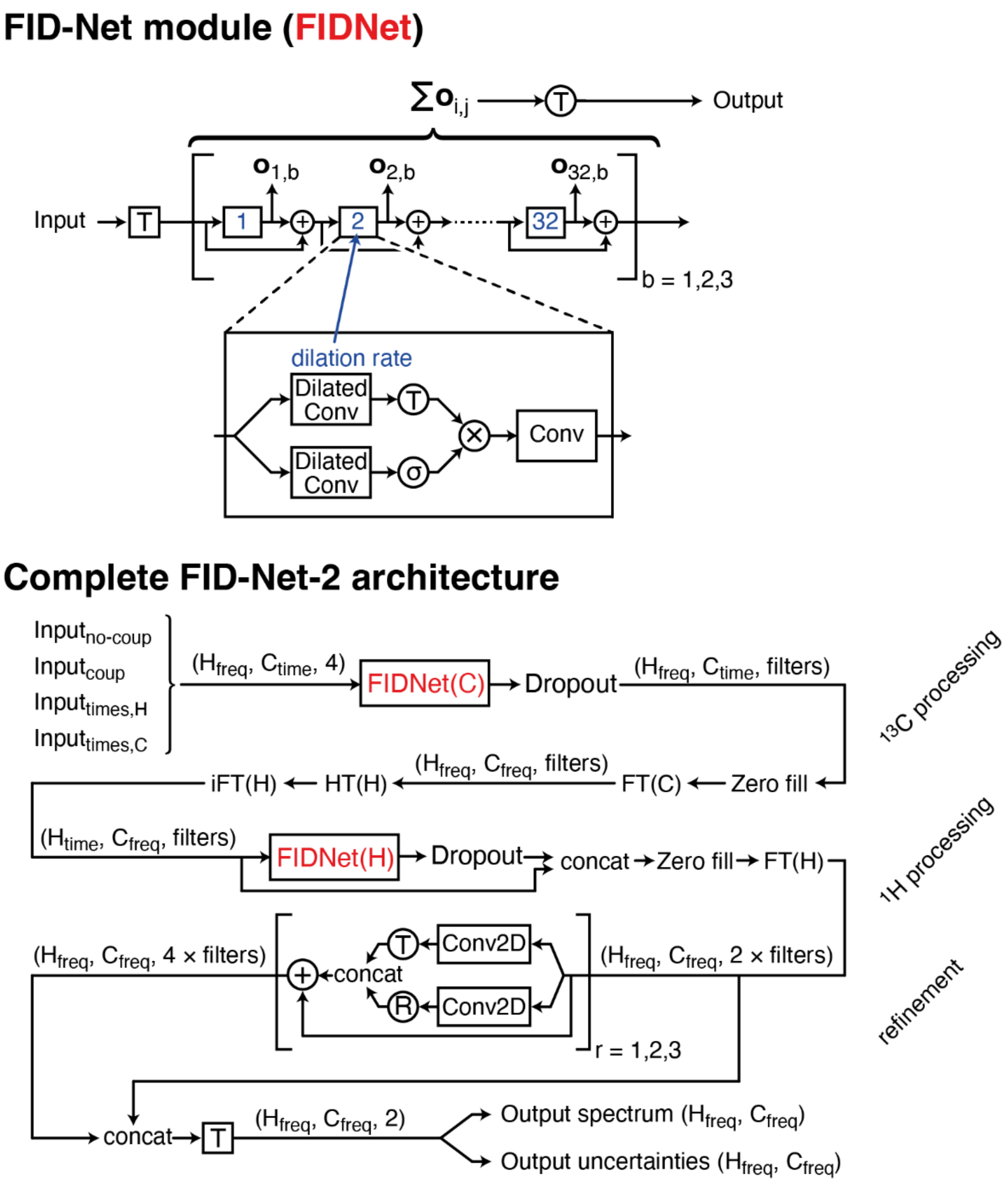

Figure S1: Summary of the FID-Net-2 Network architecture. (**a**) the FID-Net block, which is very similar to the FID-Net architecture published previously (*31*). (**b**) The FID-Net-2 architecture, which consists of mainly three parts, (*i*) a transformation of the ^{13}C dimension with an FID-Net module, (*ii*) a transformation of the ^{1}H dimension with an FID-Net module, and (*iii*) a refinement. Circles denote elementwise transformations: R: rectified linear unit, T: tanh(x) operation, σ: sigmoidal 1/(exp($-x$) +1) operation, +: summation, ×: multiplication. These elementwise operations do not include weights to be optimised. Rectangles denote layers with trainable weights: Conv: Convolutional layer, Conv2D: two-dimensional convolutional layer, T: a dense linear layer with tanh(x) activation function. The dropout rate used for the Dropout layers was 10% and was only applied during training. FT donate a Fourier transform, iFT is an inverse Fourier transform, and HT is a Hilbert transformation.

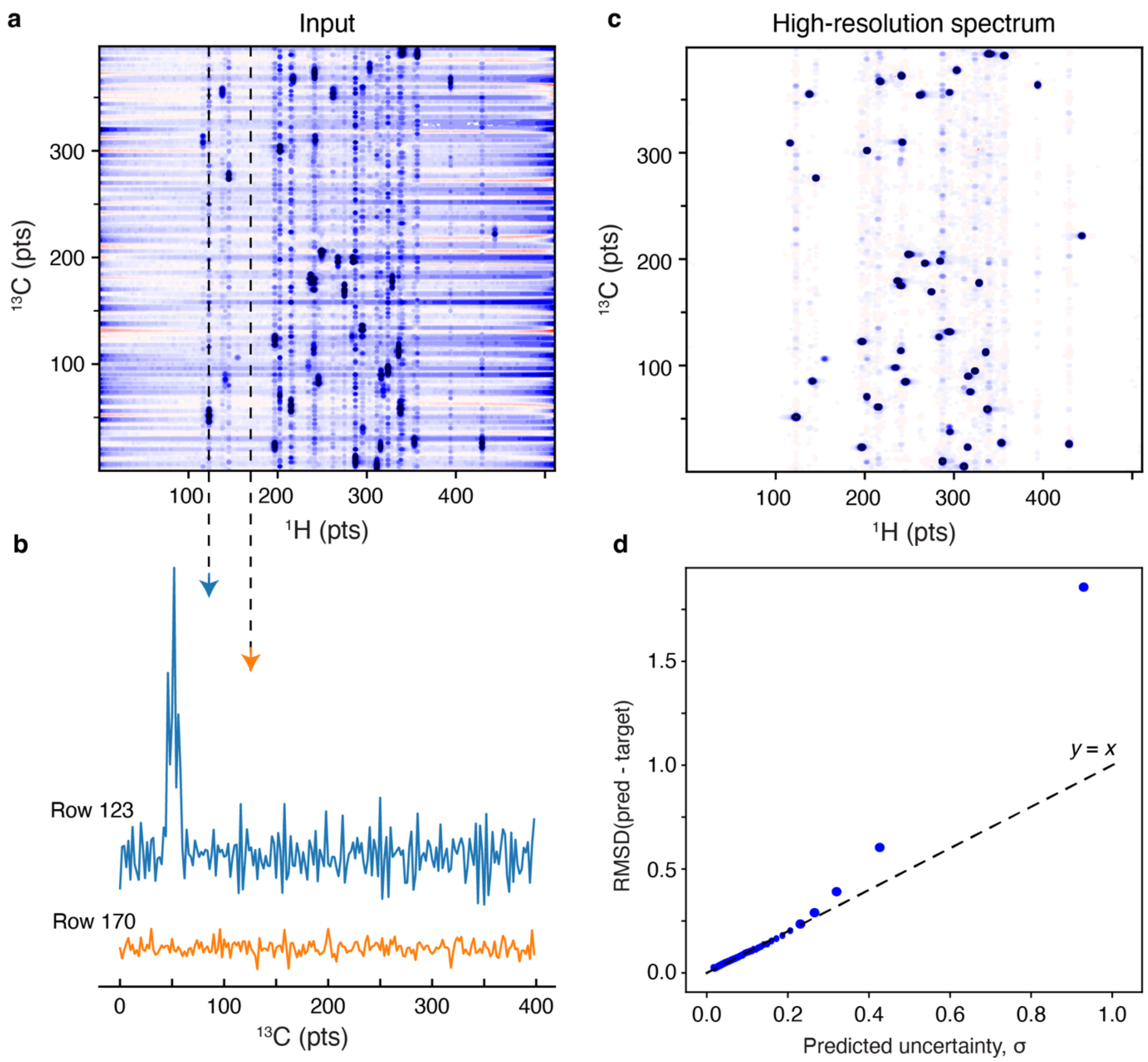

Figure S2: Assessing the performance of FID-Net-2 in the presence of t_1-noise using synthetic data. (**a**) A representative simulated spectrum, corresponding to a 20 kDa protein at 298K. On average 50 peaks are present with transverse relaxation rates of 45 s^{-1} in both the ^{1}H and ^{13}C dimension. The t_1-noise was simulated by adding Gaussian noise to the t_1 evolution time, in this case, $t_1(n) = n/\text{SW} \times \mathcal{N}(1, 0.005)$, where SW is the sweep width in Hz and $\mathcal{N}(\mu, \sigma)$ is a normal distribution with mean μ and standard deviation σ. (**b**) Slices from the spectrum in **a**, at a position where a cross-peak is present (row 123) and at a position where only noise is expected (row 170). (**c**) Spectrum obtained after processing with the FID-Net-2. Although the DNN was not trained on spectra containing t_1 noise, it is seen that the processing is robust, albeit the result not being as good as processing spectra without t_1 noise. (**d**) Predicted uncertainties *v.s.* calculated RMSD for 10 random spectra over 200 bins. The uncertainties are underestimated when substantial t_1 noise is present, however, the processing with FID-Net-2 is general robust.

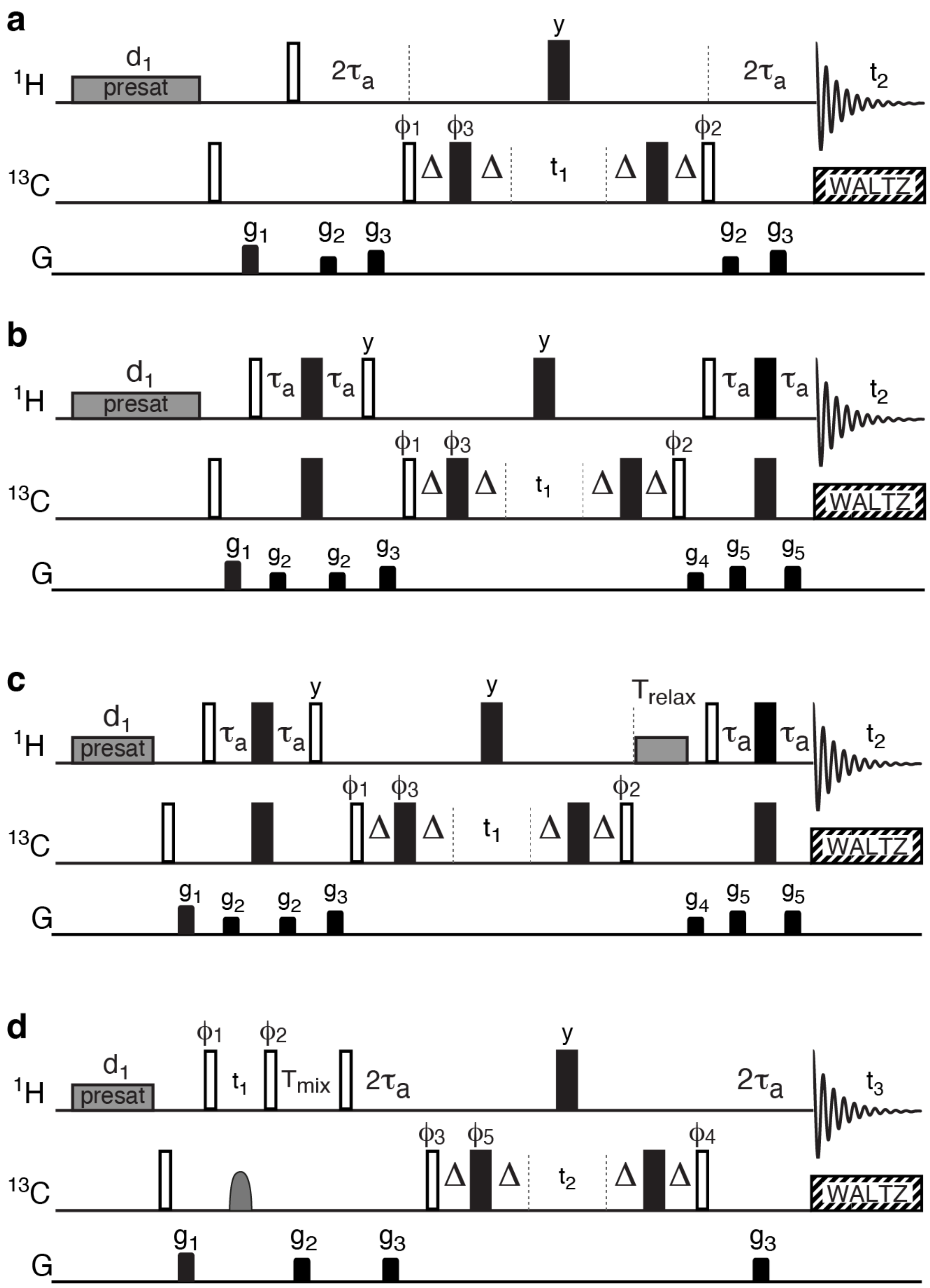

Figure S3: Pulse sequences used to generate ^{13}C-^{1}H correlation spectra for FID-Net-2 processing. Common to all sequences, (**a-d**), is that black bars represent 180° pulses, whereas open bars represent 90° non-selective pulses, that are all applied at the highest available field. The ^{1}H carrier is placed on the H_2O signal (~ 4.77 ppm) and the ^{13}C carrier placed in the middle of the aromatic region (122 ppm relative to TMS). The delays used are $\tau_a = 1/(4\ J_{CH}(\text{aro})\ ) = 1.4$ ms, whereas two planes are recorded one with $\Delta = 0.0$ and another with $\Delta = 2.3/4$ ms. Pre-saturation of the H_2O solvent signal is achieved with a 35 Hz pulse during the recovery delay d_1. The phase cycle for (**a-c**) is ϕ_1: *x*, *-x*; ϕ_2: 4(*x*), 4(*-x*); ϕ_3: *x*, *-x*, *y*, *-y*, *-x*, *x*, *-y*, *y*; ϕ_{rec}: *x*, *-x*, *-x*, *x*, *-x*, *x*, *x*, *-x*. Frequency discrimination in t_1 is obtained by States-

TPPI (*53*) of the phase ϕ_1 (States) and ϕ_2 (TPPI). (**a**) HMQC-type 2D ^{13}C-^{1}H correlation map. Gradients of 0.5 ms are represented by black rectangles and applied with strength of g_1: 3.4 G/cm, g_2: 0.8 G/cm, g_3: 1.3 G/cm. (**b**) HSQC-type 2D ^{13}C-^{1}H correlation map. Gradients of 0.5 ms are represented by black rectangles and applied with strength of g_1: 3.4 G/cm, g_2: 0.8 G/cm, g_3: 4.5 G/cm, g_4: 6.1 G/cm, g_5: 1.2 G/cm. (**c**) HSQC longitudinal exchange experiment. Gradients of 0.5 ms are represented by black rectangles and applied with strength of g_1: 3.4 G/cm, g_2: 0.8 G/cm, g_3: 4.5 G/cm, g_4: 6.1 G/cm, g_5: 1.2 G/cm. (**d**) ^{1}H-^{13}C-^{1}H NOESY experiment. The gray shaped pulse is an adiabatic inversion pulse, shape Crp80,0.5,20.1, which is applied for 500 μs and leading to inversion of a ±20 kHz frequency range. The phase cycle is ϕ_1: *x*; ϕ_2: 2(*x*), 2(-*x*); ϕ_3: *x*, -*x*; ϕ_1: *x*; ϕ_5: *x, -x, y, -y, -x, x, -y, y*; ϕ_{rec}: *x, -x, -x, x, -x, x, x, -x*. Frequency discrimination in t_1 is obtained by States-TPPI (*53*) of the phase ϕ_1 (States) and ϕ_2 (TPPI). Frequency discrimination in t_2 is obtained by States-TPPI of the phase ϕ_3 (States) and ϕ_4 (TPPI). Gradients of 0.2 ms are represented by black rectangles and applied with strength of g_1: 3.4 G/cm, g_2: 0.8 G/cm, g_3: 4.0 G/cm.

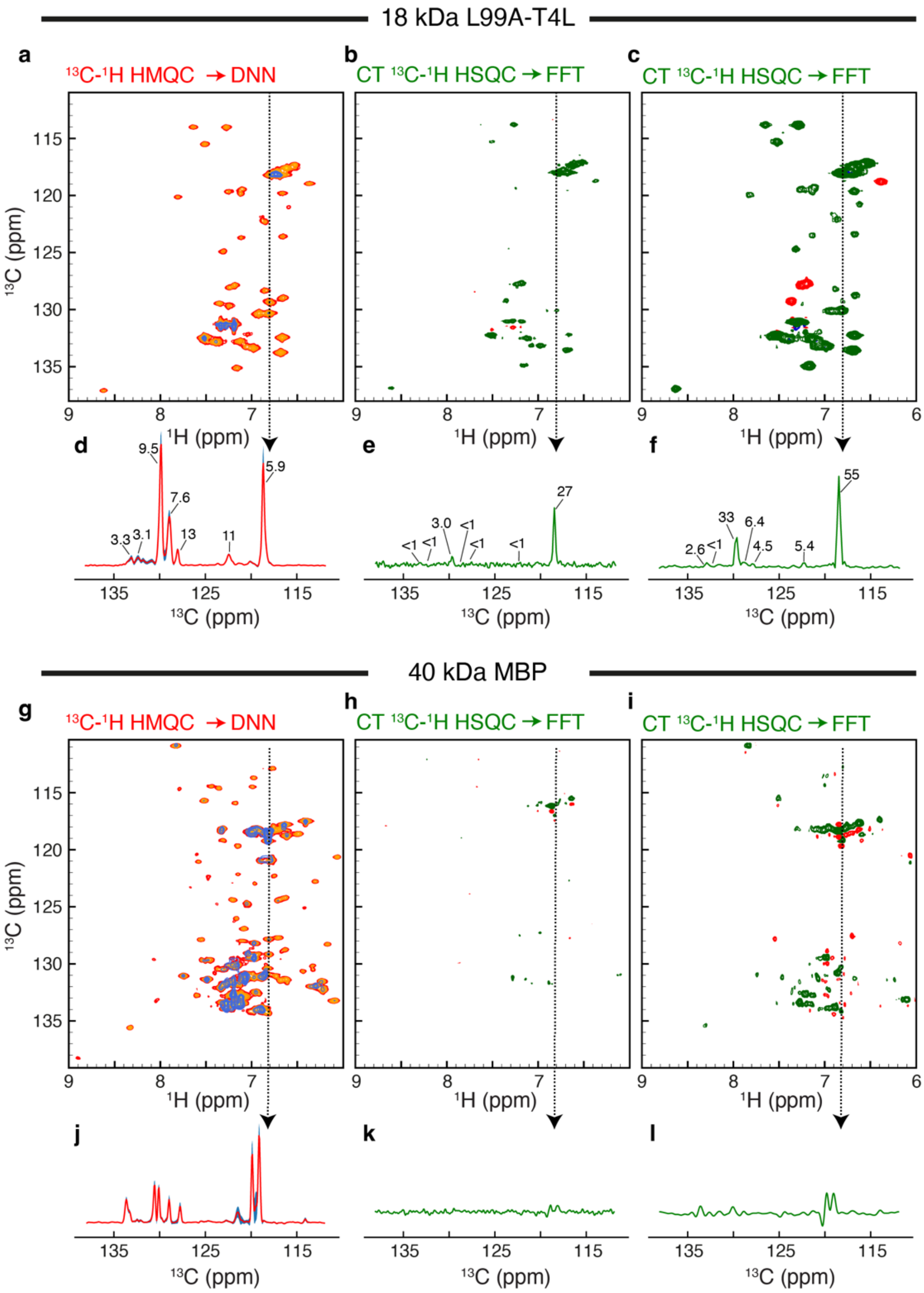

Figure S4: Comparison of FID-Net-2 transformed spectra with constant-time ^{13}C-^{1}H correlation maps. (**a**) FID-Net-2 transformed ^{13}C-^{1}H HMQC spectrum (red-yellow) along with the uncertainties (blue) of L99A-T4L (~1 mM) with 8 scans. (**b**) Constant-time ^{13}C-^{1}H HSQC recorded with a constant-time delay of 30.4 ms and with 16 scans (**c**) Constant-time ^{13}C-^{1}H HSQC recorded with a constant-time delay of 15.2 ms and 16 scans. Cross-peaks with negative intensity (red) stem from ^{13}C nuclei only scalar coupled to one adjacent ^{13}C. (**d-f**) Representative 1D slices, with the signal-to-noise for the observed peaks annotated. The signal-to-noise for the FID-Net-2 spectrum (**d**) was calculated by taking the ratio of the peak intensity and the predicted uncertainty at that position. (**g**) FID-Net-2 transformed ^{13}C-^{1}H HMQC spectrum (red-yellow) along with the uncertainties (blue) of MBP (~0.4 mM) with 32 scans. (**h**) Constant-time ^{13}C-^{1}H HSQC recorded with a constant-time delay of 30.4 ms and with 64 scans (**i**) Constant-time ^{13}C-^{1}H HSQC recorded with a constant-time delay of 15.2 ms and 140 scans. Acquisition time for each of the experiments in **g**-to-**i** were the same. (**j-l**) Representative 1D slices. All

spectra were recorded at a static magnetic field of 16.4 T (700 MHz) and at a temperature of 278K (L99A-T4L) or 310K (MBP).

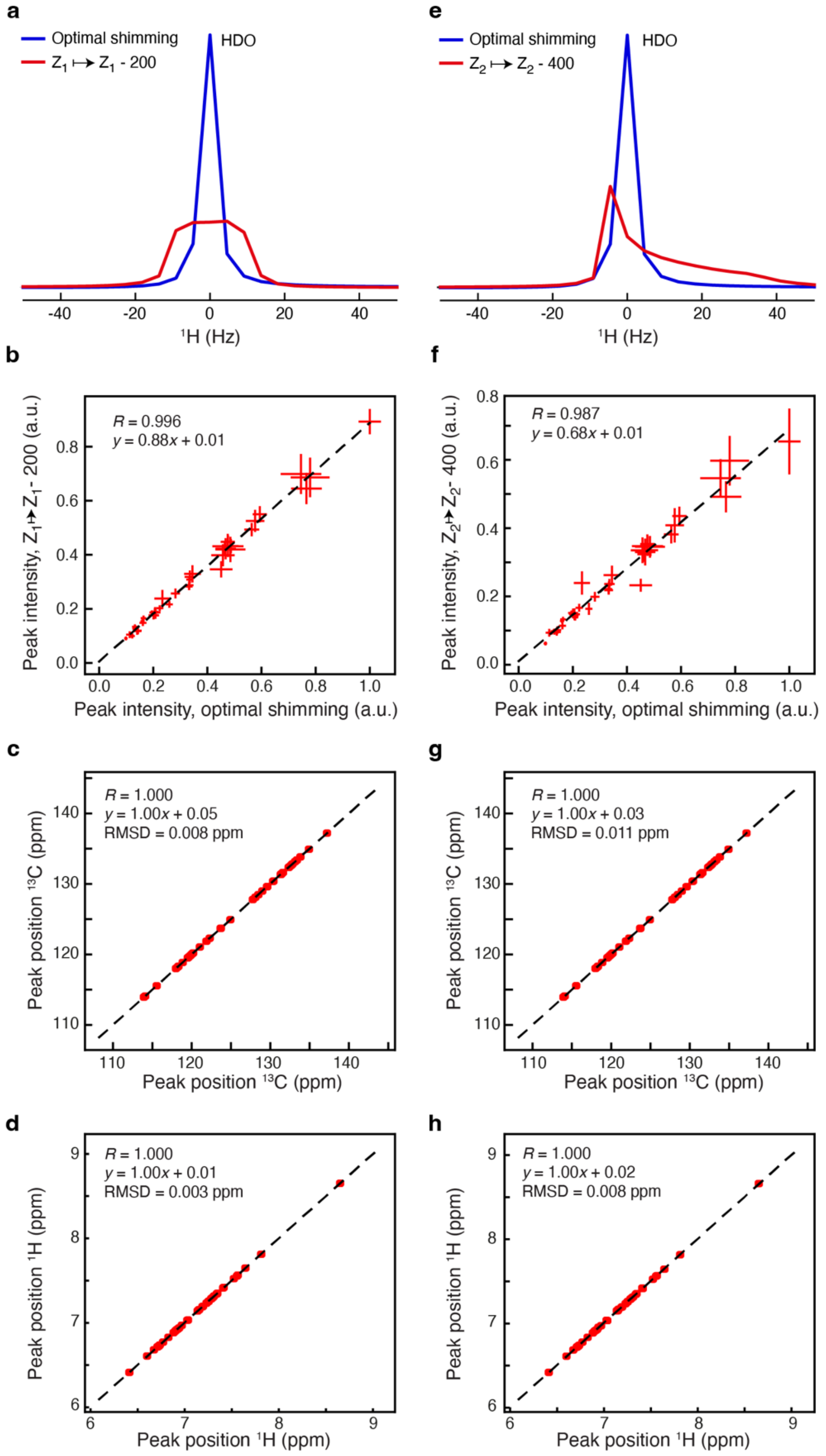

Figure S5: Assessing the performance of FID-Net-2 on datasets collected with poor shims. The assessment is carried out on L99A-T4L on data recorded at 600 MHz and a temperature of 298K. (**a,e**) One-dimensional spectra with optimal and poor shimming, when the Z_1 (**a**) or Z_2 (**e**) shims have been offset. (**b,c,d**) Assessment of peak-intensities (**b**), ^{13}C peak positions (**c**), and ^{1}H peak positions (**d**) when the Z_1 shim has been offset. (**f,g,h**) Assessment of peak-intensities (**f**), ^{13}C peak positions (**g**), and ^{1}H peak positions (**h**) when the Z_2 shim has been offset. It is important to note that the DNN was only

trained on optimally shimmed synthetic spectra and this assessment therefore represents an application to data that are substantially outside the training dataset.

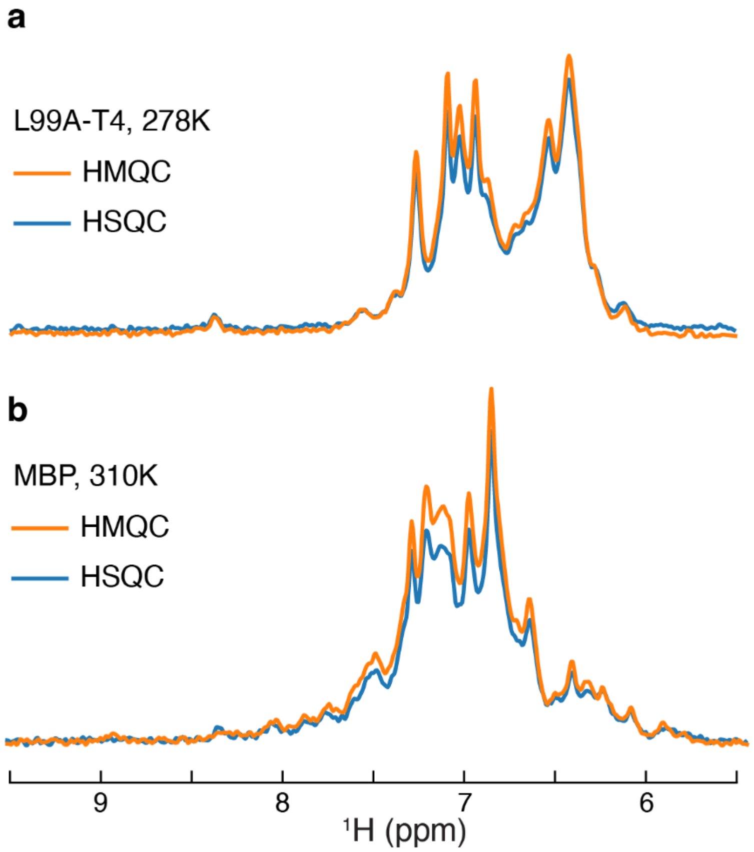

Figure S6: Sensitivity of ^{13}C-^{1}H HSQC versus ^{13}C-^{1}H HMQC. (a) Comparison of a one-dimensional ^{1}H spectra, ^{13}C(t_1) = 0 s, of L99A-T4L recorded at 700 MHz, at 278K and using a HSQC-type (blue) and HMQC-type (orange). (**b)** Comparison of a one-dimensional ^{1}H spectra, ^{13}C(t_1) = 0 s, of MBP recorded at 700 MHz, at 310K and using a HSQC-type (blue) and HMQC-type (orange). In both cases, the HMQC-type spectra are slightly more sensitive.

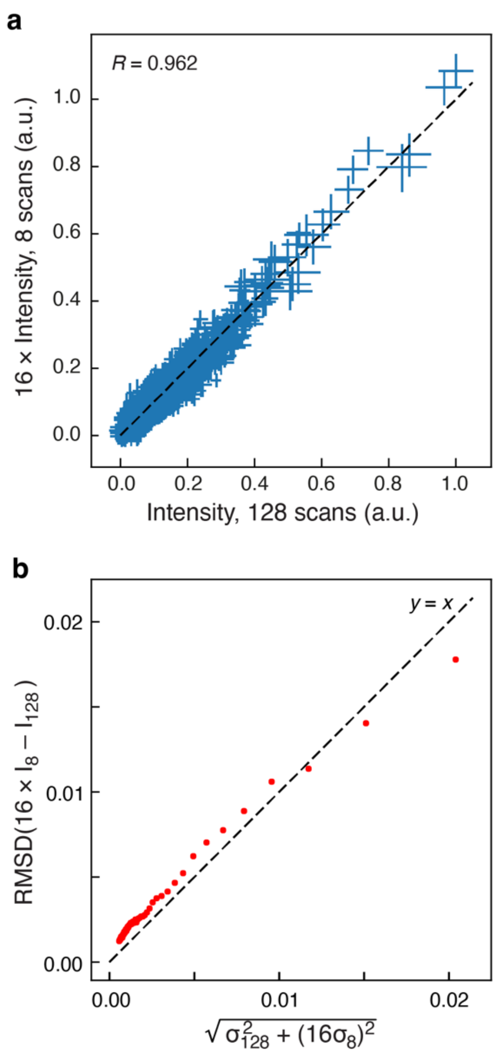

Figure S7: Comparing the performance of FID-Net-2 on datasets with differing signal to noise. Two spectra of MBP (Figure 5) were recorded with 8 scans and 128 scans. Thus, the two spectra were recorded for 1.2 h and 20h, respectively. Subsequently the two spectra were processed with FID-Net-2 network, and the results compared point-by-point between the two spectra. (**a**) Comparison of all spectral values over the 512 pts (^{1}H) × 400 pts (^{13}C). Vertical and horizontal bars represent the uncertainties predicted by the DNN and *R* is the Pearson coefficient of correlation. (**b**) Comparison of differences between the two spectra, RMSD, and the estimated uncertainties. It is seen that the DNN predicts reliable uncertainties, even in this case where the two spectra are obtained with 4 times different signal-to-noise. The uncertainty comparison is calculated over 200 linearly spaced bins.

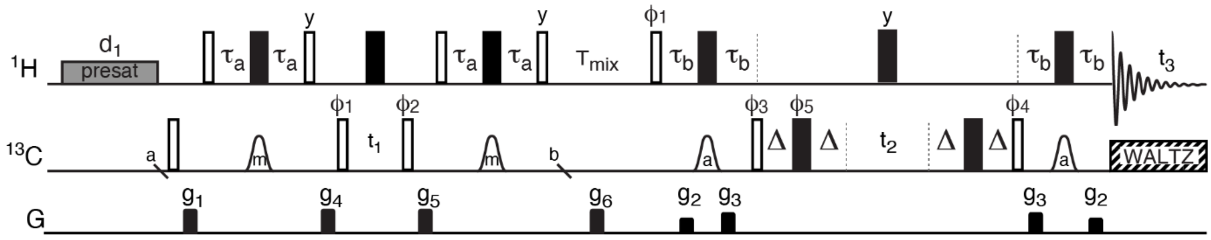

Figure S8: Pulse sequences to obtain methyl ($^{13}CH_3$) to aromatic ^{13}C-^{1}H through-space NOEs. Black bars represent 180° while open bars represent 90° non-selective pulses that are applied at the highest available field. The pre-saturation ^{1}H pulse was applied on the H_2O solvent signal using a field of 35 Hz. Open bell-shaped pulses represent frequency-selective 180° pulses using a Re-BURP shape (*52*), shaped pulses with annotation 'm' are centred at the ^{13}C methyl region (16 ppm), whereas shaped annotated with 'a' are centred at the aromatic region (122 ppm). The length of the shaped pulses were 0.95 ms (700 MHz). The ^{1}H carrier was placed on the H_2O signal, whereas the ^{13}C carrier was at 16 ppm (methyl region) between *a* and *b*, and otherwise at 122 ppm (aromatic region). The following delays are used: $\tau_a = 1/(4\ J_{CH}(\text{methyl})) = 2$ ms, $\tau_b = 1/(4\ J_{CH}(\text{aro})) = 1.4$ ms, $\Delta = 0.0$ and 2.3 ms, T_{mix} is the NOESY mixing time. Gradients of 0.2 ms are represented by black rectangles and applied with strength of g_1: 3.4 G/cm, g_2: 0.8 G/cm, g_3: 4.4 G/cm, g_4: 3.8 G/cm, g_5: 6.3 G/cm, g_6: 2.2 G/cm. The phase cycle is ϕ_1: *x*; ϕ_2: 2(*x*), 2(-*x*); ϕ_3: *x*, -*x*; ϕ_4: *x*; ϕ_5: *x*, -*x*, *y*, -*y*, -*x*, *x*, -*y*, *y*; ϕ_{rec}: *x*, -*x*, -*x*, *x*, -*x*, *x*, *x*, -*x*. Frequency discrimination in t_1 is obtained by States-TPPI (*53*) of the phase ϕ_1 (States) and ϕ_2 (TPPI). Frequency discrimination in t_2 is obtained by States-TPPI of the phase ϕ_3 (States) and ϕ_4 (TPPI).

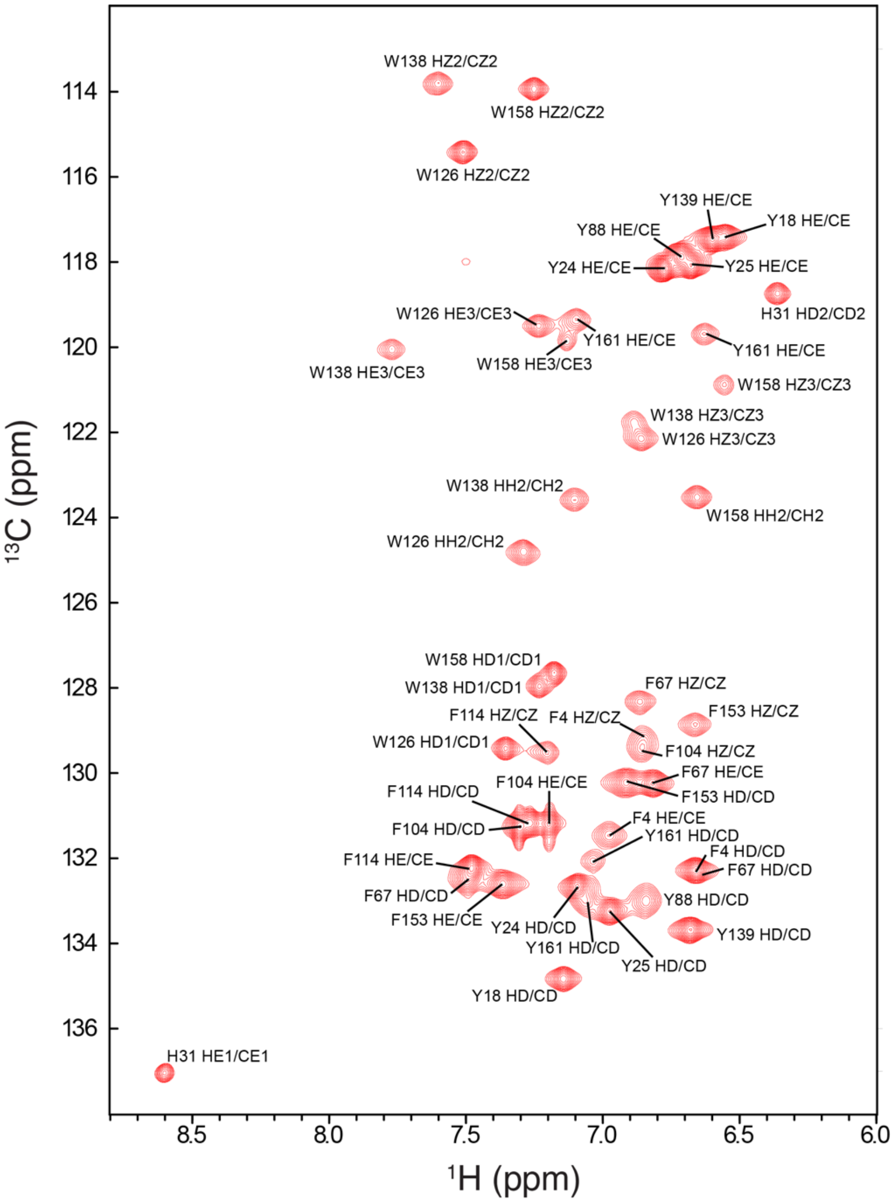

Figure S9: Assignment of the FID-Net-2 processed aromatic ^{13}C-^{1}H HSQC spectrum of L99A-T4L (700 MHz; 298K). Chemical shift assignments were obtained using the ^{1}H-^{13}C-^{1}H NOESY-HSQC and ^{13}C-^{13}C-^{1}H HSQC-NOESY-HSQC spectra described in Fig. 4.

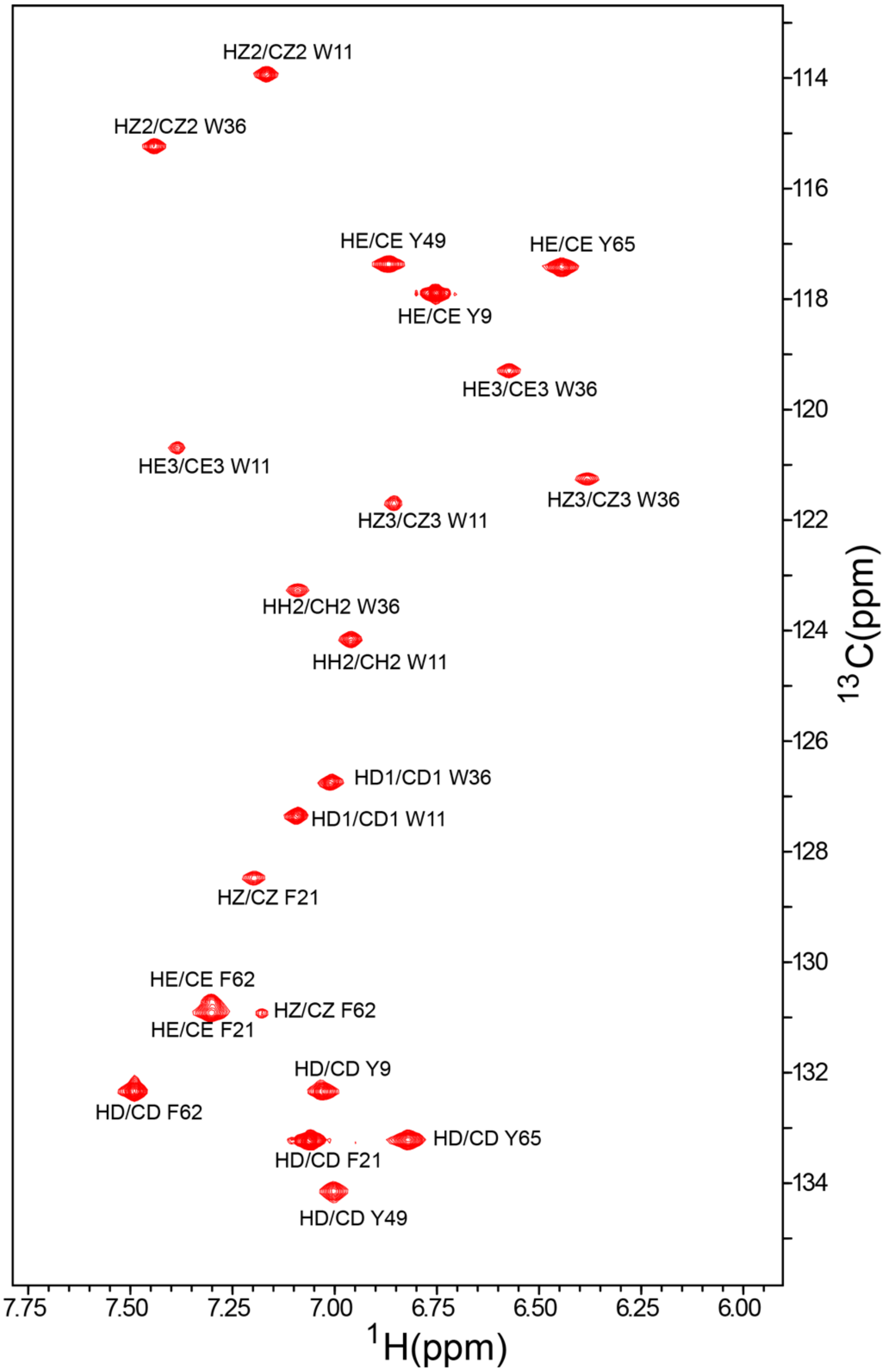

Figure S10: The FID-Net-2 processed aromatic ^{13}C-^{1}H correlation map of the A39F FF domain (700 MHz; 274 K). Assignments were obtained using the ^{1}H-^{13}C-^{1}H NOESY-HSQC and ^{13}C-^{13}C-^{1}H HSQC-NOESY-HSQC spectra.

REFERENCES AND NOTES

1. T. R. Alderson, L. E. Kay, NMR spectroscopy captures the essential role of dynamics in regulating biomolecular function. *Cell* **184**, 577–595 (2021).

2. A. G. Palmer III, Enzyme dynamics from NMR spectroscopy. *Acc. Chem. Res.* **48**, 457–465 (2015).

3. T. Xie, T. Saleh, P. Rossi, C. G. Kalodimos, Conformational states dynamically populated by a kinase determine its function. *Science* **370**, eabc2754 (2020).

4. L. Mariño Pérez, F. S. Ielasi, L. M. Bessa, D. Maurin, J. Kragelj, M. Blackledge, N. Salvi, G. Bouvignies, A. Palencia, M. R. Jensen, Visualizing protein breathing motions associated with aromatic ring flipping. *Nature* **602**, 695–700 (2022).

5. J. B. Stiller, R. Otten, D. Häussinger, P. S. Rieder, D. L. Theobald, D. Kern, Structure determination of high-energy states in a dynamic protein ensemble. *Nature* **603**, 528–535 (2022).

6. K. Madhurima, B. Nandi, S. Munshi, A. N. Naganathan, A. Sekhar, Functional regulation of an intrinsically disordered protein via a conformationally excited state. *Sci. Adv.* **9**, eadh4591 (2023).

7. V. K. Shukla, L. Siemons, D. F. Hansen, Intrinsic structural dynamics dictate enzymatic activity and inhibition. *Proc. Natl. Acad. Sci. U.S.A.* **120**, e2310910120 (2023).

8. H. W. Mackenzie, D. F. Hansen, Arginine side-chain hydrogen exchange: Quantifying arginine side-chain interactions in solution. *ChemPhysChem* **20**, 252–259 (2019).

9. A. Ceccon, V. Tugarinov, F. Torricella, G. M. Clore, Quantitative NMR analysis of the kinetics of prenucleation oligomerization and aggregation of pathogenic huntingtin exon-1 protein. *Proc. Natl. Acad. Sci. U.S.A.* **119**, e2207690119 (2022).

10. S. Guseva, V. Schnapka, W. Adamski, D. Maurin, R. W. H. Ruigrok, N. Salvi, M. Blackledge, Liquid–liquid phase separation modifies the dynamic properties of intrinsically disordered proteins. *J. Am. Chem. Soc.* **145**, 10548–10563 (2023).

11. S. Vahidi, Z. A. Ripstein, J. B. Juravsky, E. Rennella, A. L. Goldberg, A. K. Mittermaier, J. L. Rubinstein, L. E. Kay, An allosteric switch regulates Mycobacterium tuberculosis ClpP1P2 protease function as established by cryo-EM and methyl-TROSY NMR. *Proc. Natl. Acad. Sci. U.S.A.* **117**, 5895–5906 (2020).

12. V. K. Shukla, G. T. Heller, D. F. Hansen, Biomolecular NMR spectroscopy in the era of artificial intelligence. *Structure* **31**, 1360–1374 (2023).

13. J. Jumper, R. Evans, A. Pritzel, T. Green, M. Figurnov, O. Ronneberger, K. Tunyasuvunakool, R. Bates, A. Žídek, A. Potapenko, A. Bridgland, C. Meyer, S. A. A. Kohl, A. J. Ballard, A. Cowie, B. Romera-Paredes, S. Nikolov, R. Jain, J. Adler, T. Back, S. Petersen, D. Reiman, E. Clancy, M. Zielinski, M. Steinegger, M. Pacholska, T. Berghammer, S. Bodenstein, D. Silver, O. Vinyals, A. W. Senior, K. Kavukcuoglu, P. Kohli, D. Hassabis, Highly accurate protein structure prediction with AlphaFold. *Nature* **596**, 583–589 (2021).

14. K. H. Gardner, L. E. Kay, The use of ^{2}H, ^{13}C, ^{15}N multidimensional NMR to study the structure and dynamics of proteins. *Annu. Rev. Biophys. Biomol. Struct.* **27**, 357–406 (1998).

15. K. Pervushin, R. Riek, G. Wider, K. Wüthrich, Attenuated T_2 relaxation by mutual cancellation of dipole-dipole coupling and chemical shift anisotropy indicates an avenue to NMR structures of very large biological macromolecules in solution. *Proc. Natl. Acad. Sci. U.S.A.* **94**, 12366–12371 (1997).

16. V. Tugarinov, P. M. Hwang, J. E. Ollerenshaw, L. E. Kay, Cross-correlated relaxation enhanced ^{1}H–^{13}C NMR spectroscopy of methyl groups in very high molecular weight proteins and protein complexes. *J. Am. Chem. Soc.* **125**, 10420–10428 (2003).

17. N. D. Werbeck, J. Kirkpatrick, D. F. Hansen, Probing arginine side-chains and their dynamics with carbon-detected NMR spectroscopy: Application to the 42 kDa human histone deacetylase 8 at high pH. *Angew. Chem. Int. Ed. Engl.* **52**, 3145–3147 (2013).

18. R. B. Pritchard, D. F. Hansen, Characterising side chains in large proteins by protonless 13C-detected NMR spectroscopy. *Nat. Commun.* **10**, 1747 (2019).

19. A. Esadze, C. Chen, L. Zandarashvili, S. Roy, B. M. Pettitt, J. Iwahara, Changes in conformational dynamics of basic side chains upon protein–DNA association. *Nucleic Acids Res.* **44**, 6961–6970 (2016).

20. K. A. Stafford, F. Ferrage, J.-H. Cho, A. G. Palmer III, Side chain dynamics of carboxyl and carbonyl groups in the catalytic function of Escherichia coli ribonuclease H. *J. Am. Chem. Soc.* **135**, 18024–18027 (2013).

21. J. Santoro, G. C. King, A constant-time 2D overbodenhausen experiment for inverse correlation of isotopically enriched species. *J. Magn. Reson.* **97**, 202–207 (1992).

22. G. W. Vuister, A. Bax, Resolution enhancement and spectral editing of uniformly ^{13}C-enriched proteins by homonuclear broadband ^{13}C decoupling. *J. Magn. Reson.* **98**, 428–435 (1992).

23. K. Teilum, U. Brath, P. Lundström, M. Akke, Biosynthetic ^{13}C labeling of aromatic side chains in proteins for NMR relaxation measurements. *J. Am. Chem. Soc.* **128**, 2506–2507 (2006).

24. M. Akke, U. Weininger, NMR studies of aromatic ring flips to probe conformational fluctuations in proteins. *J. Phys. Chem. B* **127**, 591–599 (2023).

25. U. Weininger, Optimal isotope labeling of aromatic amino acid side chains for NMR studies of protein dynamics. *Methods Enzymol.* **614**, 67–86 (2019).

26. B. M. Young, P. Rossi, P. J. Slavish, Y. Cui, M. Sowaileh, J. Das, C. G. Kalodimos, Z. Rankovic, Synthesis of isotopically labeled, spin-isolated tyrosine and phenylalanine for protein NMR applications. *Org. Lett.* **23**, 6288–6292 (2021).

27. Y. LeCun, Y. Bengio, G. Hinton, Deep learning. *Nature* **521**, 436–444 (2015).

28. M. Baek, F. DiMaio, I. Anishchenko, J. Dauparas, S. Ovchinnikov, G. R. Lee, J. Wang, Q. Cong, L. N. Kinch, R. D. Schaeffer, C. Millán, H. Park, C. Adams, C. R. Glassman, A. DeGiovanni, J. H. Pereira, A. V. Rodrigues, A. A. van Dijk, A. C. Ebrecht, D. J. Opperman, T. Sagmeister, C. Buhlheller, T. Pavkov-Keller, M. K. Rathinaswamy, U. Dalwadi, C. K. Yip, J. E. Burke, K. C.

Garcia, N. V. Grishin, P. D. Adams, R. J. Read, D. Baker, Accurate prediction of protein structures and interactions using a three-track neural network. *Science* **373**, 871–876 (2021).

29. S. G. Worswick, J. A. Spencer, G. Jeschke, I. Kuprov, Deep neural network processing of DEER data. *Sci. Adv.* **4**, eaat5218 (2018).

30. X. Qu, Y. Huang, H. Lu, T. Qiu, D. Guo, T. Agback, V. Orekhov, Z. Chen, Accelerated nuclear magnetic resonance spectroscopy with deep learning. *Angew. Chem. Int. Ed. Engl.* **59**, 10297–10300 (2020).

31. G. Karunanithy, D. F. Hansen, FID-Net: A versatile deep neural network architecture for NMR spectral reconstruction and virtual decoupling. *J. Biomol. NMR* **75**, 179–191 (2021).

32. G. Karunanithy, H. W. Mackenzie, D. F. Hansen, Virtual homonuclear decoupling in direct detection nuclear magnetic resonance experiments using deep neural networks. *J. Am. Chem. Soc.* **143**, 16935–16942 (2021).

33. D.-W. Li, A. L. Hansen, C. Yuan, L. Bruschweiler-Li, R. Brüschweiler, DEEP picker is a deep neural network for accurate deconvolution of complex two-dimensional NMR spectra. *Nat. Commun.* **12**, 5229 (2021).

34. D. F. Hansen, Using deep neural networks to reconstruct non-uniformly sampled NMR spectra. *J. Biomol. NMR* **73**, 577–585 (2019).

35. G. Karunanithy, V. K. Shukla, D. F. Hansen, Solution-state methyl NMR spectroscopy of large non-deuterated proteins enabled by deep neural networks. *Nat. Commun.* **15**, 5073 (2024); https://doi.org/10.1038/s41467-024-49378-8.

36. A. E. Eriksson, W. A. Baase, J. A. Wozniak, B. W. Matthews, A cavity-containing mutant of T4 lysozyme is stabilized by buried benzene. *Nature* **355**, 371–373 (1992).

37. Y. Pustovalova, F. Delaglio, D. L. Craft, H. Arthanari, A. Bax, M. Billeter, M. J. Bostock, H. Dashti, D. F. Hansen, S. G. Hyberts, B. A. Johnson, K. Kazimierczuk, H. Lu, M. Maciejewski, T. M. Miljenović, M. Mobli, D. Nietlispach, V. Orekhov, R. Powers, X. Qu, S. A. Robson, D. Rovnyak, G. Wagner, J. Ying, M. Zambrello, J. C. Hoch, D. L. Donoho, A. D.

Schuyler, NUScon: A community-driven platform for quantitative evaluation of nonuniform sampling in NMR. *Magn. Reson.* **2**, 843–861 (2021).

38. G. Bouvignies, P. Vallurupalli, D. F. Hansen, B. E. Correia, O. Lange, A. Bah, R. M. Vernon, F. W. Dahlquist, D. Baker, L. E. Kay, Solution structure of a minor and transiently formed state of a T4 lysozyme mutant. *Nature* **477**, 111–114 (2011).

39. M. Tollinger, N. R. Skrynnikov, F. A. A. Mulder, J. D. Forman-Kay, L. E. Kay, Slow dynamics in folded and unfolded states of an SH3 domain. *J. Am. Chem. Soc.* **123**, 11341–11352 (2001).

40. V. P. Tiwari, Y. Toyama, D. De, L. E. Kay, P. Vallurupalli, The A39G FF domain folds on a volcano-shaped free energy surface via separate pathways. *Proc. Natl. Acad. Sci. U.S.A.* **118**, e2115113118 (2021).

41. A. Boeszoermenyi, S. Chhabra, A. Dubey, D. L. Radeva, N. T. Burdzhiev, C. D. Chanev, O. I. Petrov, V. M. Gelev, M. Zhang, C. Anklin, H. Kovacs, G. Wagner, I. Kuprov, K. Takeuchi, H. Arthanari, Aromatic ^{19}F-^{13}C TROSY: A background-free approach to probe biomolecular structure, function, and dynamics. *Nat. Methods* **16**, 333–340 (2019).

42. L.-P. Picard, R. S. Prosser, Advances in the study of GPCRs by ^{19}F NMR. *Curr. Opin. Struct. Biol.* **69**, 169–176 (2021).

43. M. Abadi, A. Agarwal, P. Barham, E. Brevdo, Z. Chen, C. Citro, G. S. Corrado, A. Davis, J. Dean, M. Devin, S. Ghemawat, I. Goodfellow, A. Harp, G. Irving, M. Isard, R. Jozefowicz, Y. Jia, L. Kaiser, M. Kudlur, J. Levenberg, D. Mané, M. Schuster, R. Monga, S. Moore, D. Murray, C. Olah, J. Shlens, B. Steiner, I. Sutskever, K. Talwar, P. Tucker, V. Vanhoucke, V. Vasudevan, F. Viégas, O. Vinyals, P. Warden, M. Wattenberg, M. Wicke, Y. Yu, X. Zheng, TensorFlow: Large-scale machine learning on heterogeneous systems (2015); www.tensorflow.org.

44. F. Chollet, Keras (2015); https://keras.io.

45. D. P. Kingma, J. Ba, Adam: A method for stochastic optimization. arXiv:1412.6980 (2014).

46. M. W. Maciejewski, A. D. Schuyler, M. R. Gryk, I. I. Moraru, P. R. Romero, E. L. Ulrich, H. R. Eghbalnia, M. Livny, F. Delaglio, J. C. Hoch, NMRbox: A resource for biomolecular NMR computation. *Biophys. J.* **112**, 1529–1534 (2017).

47. K. H. Gardner, X. Zhang, K. Gehring, L. E. Kay, Solution NMR studies of a 42 KDa Escherichia C oli maltose binding protein/β-cyclodextrin complex: Chemical shift assignments and analysis. *J. Am. Chem. Soc.* **120**, 11738–11748 (1998).

48. F. Delaglio, S. Grzesiek, G. W. Vuister, G. Zhu, J. Pfeifer, A. Bax, Nmrpipe – A Multidimensional spectral processing system based on Unix pipes. *J. Biomol. NMR* **6**, 277–293 (1995).

49. J. J. Helmus, C. P. Jaroniec, Nmrglue: An open source Python package for the analysis of multidimensional NMR data. *J. Biomol. NMR* **55**, 355–367 (2013).

50. L. Liu, W. A. Baase, B. W. Matthews, Halogenated benzenes bound within a non-polar cavity in T4 lysozyme provide examples of I…S and I…Se halogen-bonding. *J. Mol. Biol.* **385**, 595–605 (2009).

51. H. M. McConnell, Reaction rates by nuclear magnetic resonance. *J. Chem. Phys.* **28**, 430–431 (1958).

52. H. Geen, R. Freeman, Band-selective radiofrequency pulses. *J. Magn. Reson.* **93**, 93–141 (1991).

53. D. Marion, M. Ikura, R. Tschudin, A. Bax, Rapid recording of 2D NMR spectra without phase cycling. Application to the study of hydrogen exchange in proteins. *J. Magn. Reson.* **85**, 393–399 (1989).
